# Carboxylesterase 1-mediated endocannabinoid metabolism in skin: role in melanoma progression in *BRaf*^*V600E*^/*Pten*^*−/−*^ mice

**DOI:** 10.1186/s40170-025-00378-2

**Published:** 2025-02-11

**Authors:** Veronika Morozova, Daniele Pellegata, Roch-Philippe Charles, Jürg Gertsch

**Affiliations:** Institute of Biochemistry and Molecular Medicine, University of Bern, Bern, CH-3012 Switzerland

**Keywords:** CES1, Melanoma, 2-AG, Lipidomics, Targeted Metabolomics, Chemoproteomics, Cancer, Biomarkers

## Abstract

**Background:**

Melanoma is a highly aggressive skin cancer with a poor prognosis. The endocannabinoids 2-arachidonoylgylcerol (2-AG) and anandamide have been linked to melanoma progression, though their roles remain unclear. We hypothesized that the 2-AG-arachidonate-prostaglandin axis could drive aggressive melanoma progression.

**Methods:**

The genetically engineered melanoma mouse model *B6-Tyr::CreER*^*T2*^*; BRaf*^*CA*^*; Pten*^*loxP*^ was characterized by targeted metabolomics. Functionally expressed serine hydrolases in the tumor tissue were identified by chemoproteomics. Pharmacological inhibition of carboxylesterase 1 (CES1) was achieved through chronic *in viv*o i.p. treatment with JZL184 (10 mg/kg daily), confirmed by activity-based protein profiling (ABPP) and targeted lipidomics. CES1-mediated 2-AG hydrolysis was further confirmed in radiotracer-based assays using CES1-transfected cell lines.

**Results:**

The diacylglycerol and protein kinase C activator 1-stearoyl-2-arachidonoyl-sn-glycerol (SAG) was significantly elevated in the nodular-like melanoma tissues, along with 2-AG and arachidonic acid (ARA), compared to normal skin. AEA and other *N*-acylethanolamines were decreased, while, notably, prostaglandin levels remained unchanged. Significant changes in the levels of neuromodulators and neurotransmitters, including serotonin and adenosine, were observed. Pronounced differences between serine hydrolase activity in normal skin and melanoma tissue were identified by ABPP. Intriguingly, CES1 was identified as the only 2-AG-hydrolyzing enzyme in this melanoma tissue, as MAGL and ABHD6/12 were not expressed. The MAGL inhibitor JZL184 also efficiently inhibited CES1 in vitro and in vivo*,* increasing glycerol esters and reducing tumor progression. Additionally, scRNA-seq data from previous studies revealed divergent MAGL/CES1 expression patterns across different human melanoma subtypes.

**Conclusions:**

A role of CES1 expression in skin is demonstrated for the first time. Our study suggests that 2-AG degradation to arachidonate favors melanoma progression, either reflecting the carcinogenic role of ARA or that monoacylglycerols like 2-AG and/or other CES1 substrates may exert antitumor effects, indicating that CES1 could be a potential therapeutic target. CES1 expression and high SAG, 2-AG, and ARA levels may be a signature of specific BRAF-driven malignant melanoma subtypes which are associated with discrete metabolic adaptations.

**Graphical Abstract:**

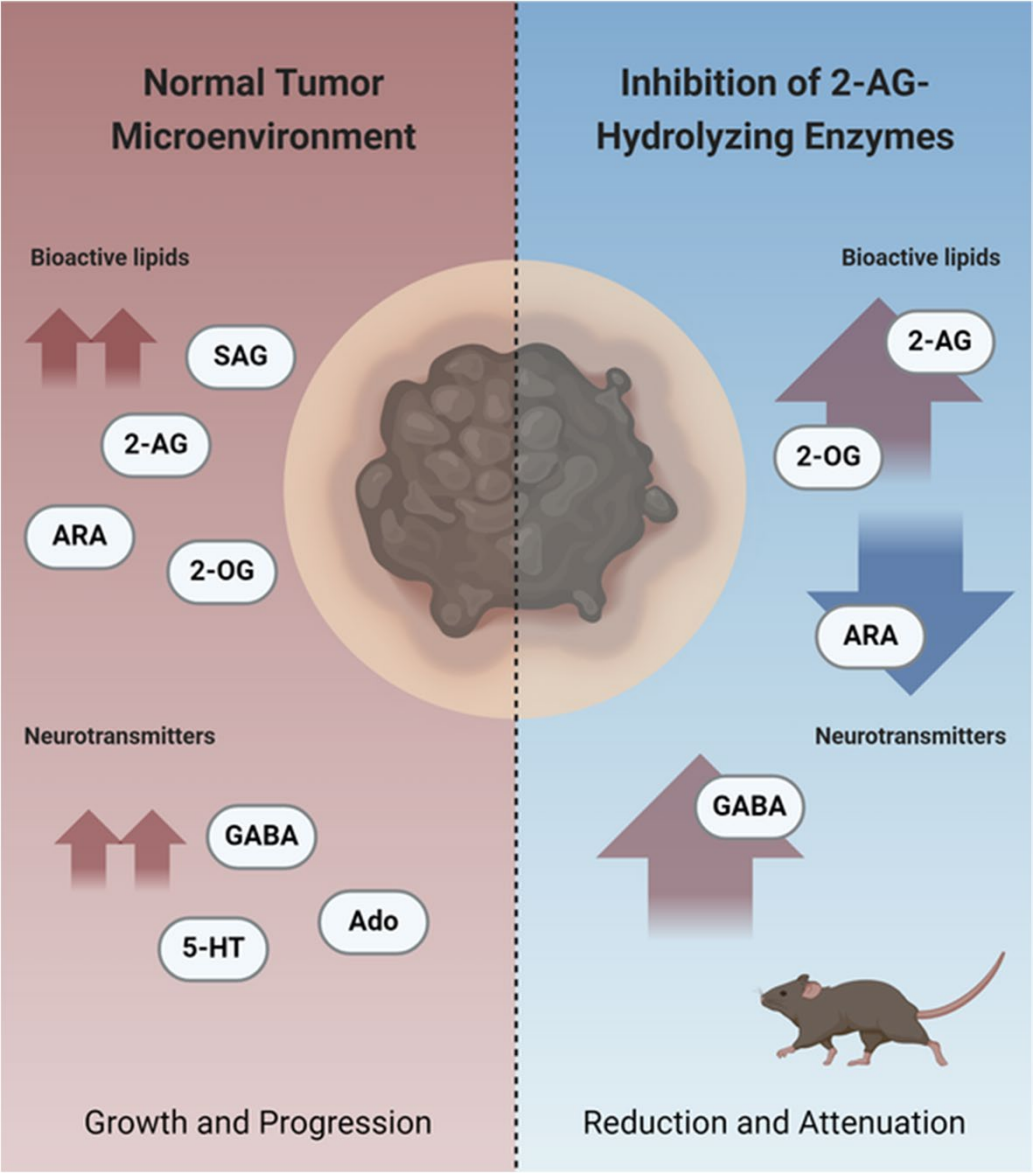

**Supplementary Information:**

The online version contains supplementary material available at 10.1186/s40170-025-00378-2.

## Introduction

Malignant melanoma, the most aggressive form of skin cancer, poses a significant challenge for therapy due to its heterogeneity, high risk for metastasis, and resistance to conventional treatments [1]. Melanomas are clinically categorized by their growth pattern (such as superficial spreading, lentigo, or nodular melanoma), anatomical location (e.g., cutaneous, mucosal, or acral melanoma), or pigmentation status (e.g., melanotic versus amelanotic melanoma) [2]. Approximately 50% of melanomas carry activating *BRAF* mutations, with over 90% being the V600E variant. *BRAF*^*V600E*^ has been linked to various mechanisms driving melanomagenesis, primarily through the dysregulated activation of downstream MEK/ERK effectors [3]. Recent advancements in treatment, particularly with targeted therapies, such as BRAF, MEK, and immune checkpoint inhibitors, have significantly improved the prognosis for patients with common subtypes of cutaneous melanoma (CM) [4, 5]. However, research and clinical trials predominantly focus on the common subtypes, resulting in a significant gap in our understanding of the molecular characteristics and effective treatment strategies for rarer melanoma variants from patients with CM who present increased metastasis and resistance to treatments [4]. This highlights the urgent need to identify molecular markers to better characterize the heterogeneity and subtypes of melanoma, which is crucial for understanding both the mechanisms behind individual treatment responses and possible resistance to targeted therapy.

**Fig. 1:**
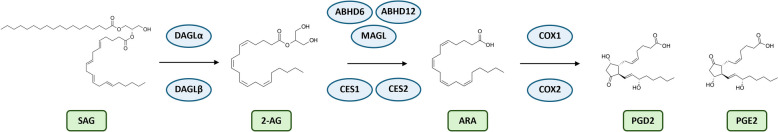
Schematic representation of the 2-AG-ARA-PGs axis. Abbreviations: 2-AG, 2-arachidonoylglycerol; ABHD6, alpha/beta-hydrolase domain-containing protein 6; ABHD12, alpha/beta-hydrolase domain-containing protein 12; ARA, arachidonic acid; CES1, carboxylesterase 1; CES2, carboxylesterase 2; COX1, Cyclooxygenase 1 COX2, Cyclooxygenase 2; DAGL α, Diacylglycerol lipase α; DAGL β, Diacylglycerol lipase β; MAGL, monoacylglycerol lipase; PGD2, prostaglandin D2; PGE2, prostaglandin E2; SAG, 1-stearoyl-2-arachidonoyl-sn-glycerol

To date, few studies have explored the role of the endocannabinoid system (ECS), particularly the endocannabinoid-prostaglandin axis, in melanoma development and progression [6, 7]. The roles of endocannabinoids in melanoma thus remain unclear, as both pro- and anti-tumorigenic effects have been described [8]. For example, high levels of arachidonic acid (ARA), the major tissue n-6 polyunsaturated fatty acid, have been implicated in promoting tumor progression through the activation of pro-inflammatory pathways, modulation of the tumor microenvironment, and enhancement of metastatic potential [9, 10]. ARA is a precursor of eicosanoids, including prostaglandins (Fig. 1) and leukotrienes, which are generated via cyclooxygenases and can contribute to cancer cell proliferation, angiogenesis, and resistance to apoptosis [11, 12]. In addition to the classical PLA_2_ pathway leading to ARA from plasma membranes, the major tissue endocannabinoid 2-arachidonoylglycerol ( 2-AG) may serve as a precursor for ARA and potentially eicosanoids (Fig. 1) [13]. Elevated levels of 2-AG have also been shown to exert antitumor effects in pancreatic cancer [14] and non-small cell lung cancer [15]. The primary tissue enzyme responsible for 2-AG degradation is monoacylglycerol lipase (MAGL) (Fig. 1) [16–19].

Notably, several other serine hydrolases can hydrolyze 2-AG in a cell- and tissue-specific manner, including alpha/beta-hydrolase domain-containing proteins 6 and 12 (ABHD6, ABHD12) [19], and carboxylesterases 1 and 2 (CES1, CES2) [20, 21]. Among these, carboxylesterases (CES), which are members of the serine hydrolase family, play a significant role by hydrolyzing carboxylic esters and amides [22, 23]. The role of CES1 in the ECS remains poorly understood.

In humans, CES1 is the predominant isoform, primarily found in the liver but also present in other tissues, including adipose tissue, lungs, and intestine [24–26]. CES1 plays an essential chemoprotective role, through the detoxification of a wide range of molecules, including narcotics (e.g., cocaine and heroin), pesticides, and other xenobiotics [27–30]. It also participates in the metabolism of several therapeutic drugs, such as clopidogrel [31] and oseltamivir [32], and promotes the conversion of different prodrugs to their active forms, including imidapril and temocapril [33]. In mice, eight Ces1 isoforms have been reported—*Ces1a*, *Ces1b*, *Ces1c*, *Ces1d*, *Ces1e*, *Ces1f*, *Ces1g*, and *Ces1h* [34], each showing a distinctive tissue distribution and substrate affinities compared to the human CES1 [22].

Importantly, CES1 plays a vital role in endogenous lipid metabolism by hydrolyzing triglycerides and cholesterol esters, regulating intracellular levels of free fatty acids and cholesterol, and maintaining lipid homeostasis [30]. Dysregulation of CES1 activity can lead to altered lipid metabolism [22, 35], contributing to pathologies such as obesity, diabetes, and atherosclerosis [36–38]. Interestingly, some studies have implicated CES1 expression in cancer pathophysiology as a biomarker and a potential therapeutic target for hepatocellular carcinoma [39–41] and colorectal cancer [42]. However, to date, the role of CES1 in skin or melanoma progression has not been reported.

**Fig. 2:**
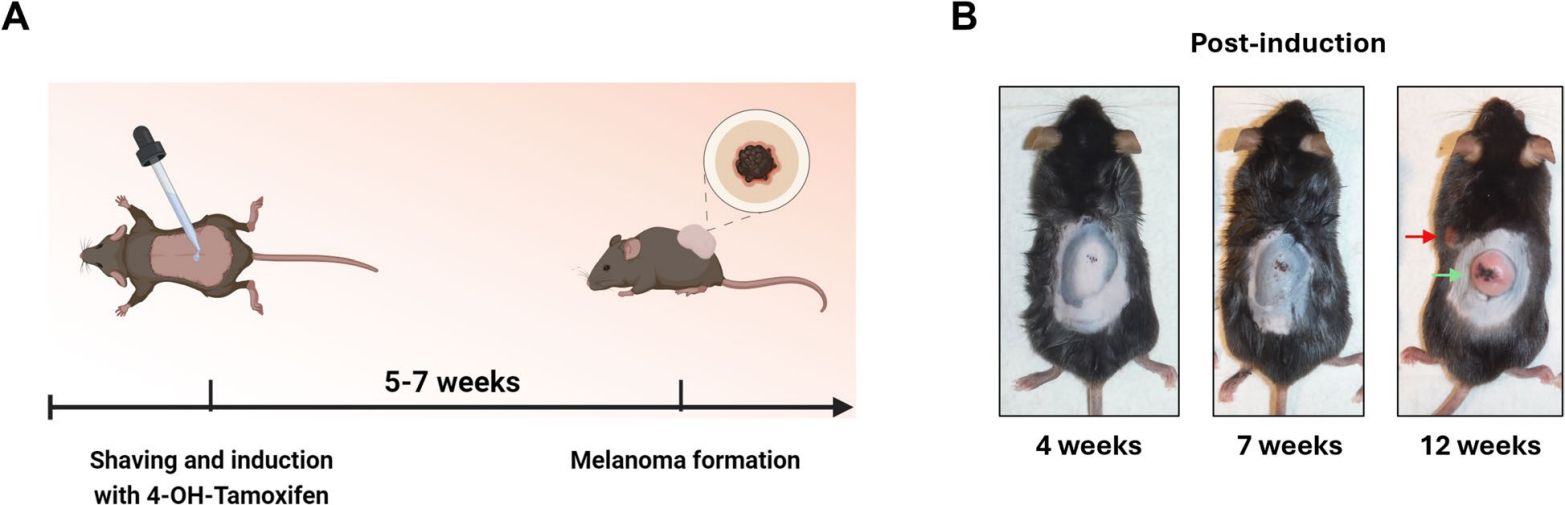
Melanoma induction in the B6-Tyr::CreER^T2^; BRaf^CA^; Pten.^loxP^ mouse model. **A** Schematic representation of the timeline for tumor induction and formation. **B** Representative images of a mouse used in this study showing tumor formation and progression after induction with 4-OHT. Green arrow indicates an induced nodular-like tumor, red arrow indicates a spontaneous tumor formed due to Cre-recombinase “leakage”

In this study, we used a genetically engineered mouse model of human malignant melanoma based on the findings of Dankort et al*.,* who in 2009 demonstrated that the *BRaf*^*V600E*^ mutation, combined with the loss of the tumor suppressor gene *Pten*, leads to the development of a highly aggressive form of nodular-like melanoma in mice, characterized by 100% penetrance, short latency and metastases in lymph nodes and lungs [43]. The model is based on the melanocyte-specific expression of *BRaf*^*V600E*^ and *Pten* deletion mediated by Cre-lox recombination under the control of the Tyrosinase-CreER^T2^ promoter. Cre recombinase is activated by the active metabolite of the pro-drug tamoxifen, 4-hydroxytamoxifen (4-OHT), which allows the Cre-lox recombination only in melanocytes and leads to the melanoma formation within 5–7 weeks (Fig. 2A) [43]. This model shows a translationally relevant genetic profile and mimics the molecular pathology of human malignant melanoma, providing a basis for studying the molecular mechanisms underlying melanoma progression and for the pre-clinical evaluation of targeted therapies.

Despite these advantages, the model presents challenges due to its aggressiveness and a high rate of spontaneous tumor formation—60 to 90% of mice develop tumors even without 4-OHT induction. This "leakiness" in Cre recombinase expression and activity leads to spontaneous activation of *Braf*^*V600E*^ and *Pten* deletion, resulting in tumorigenesis without the controlled chemical induction [44, 45]. Because the spontaneous tumors vary in consistency and cannot be reliably used for controlled experiments, they were omitted from this study (Fig. 2B). A precise induction was critical for obtaining accurate melanoma formation. The induction over a large skin area resulted in multiple tumors, complicating volume assessment during treatment. Therefore, it was crucial to minimize the induction area to avoid widespread melanoma formation. Precise application of 4-OHT to a confined area of the skin was essential, avoiding any spillage that could cause multiple tumors. Additionally, we found that in some cases, a single application of 4-OHT was insufficient for melanoma development, and repeated inductions over three consecutive days were necessary.

The present study provides novel insights into the metabolism of bioactive lipids in the context of *BRaf*^*V600E*^-driven malignant melanoma. We found that certain aggressive and metastatic nodular-like melanomas with low NAEs but high 1-stearoyl-2-arachidonoyl-sn-glycerol (SAG), 2-AG, and ARA levels can progress without MAGL and upregulate CES1 to drive the hydrolysis of 2-AG, 2-oleoylglycerol (2-OG), and other glycerol esters (Fig. 1). This finding highlights a previously underexplored compensatory mechanism that may help melanomas to sustain their growth and progression through alternative lipid metabolic pathways characterized by high SAG, 2-AG, and ARA levels and CES1/Ces1 overexpression in a subtype of *BRAF*^*V600E*^-driven malignant melanoma. We show that in the absence of MAGL, CES1 significantly contributes to the hydrolysis of 2-AG and other glycerol esters in the skin, inhibiting melanoma progression. Importantly, our in vivo experiments revealed that in tumors with elevated SAG levels, inhibiting Ces1c markedly impairs melanoma tumor growth, highlighting CES1 as a potential therapeutic target for malignant melanoma.

We identified Ces1c as the key isoform present in these nodular-like melanomas. Our in vivo experiments revealed that covalent Ces1c inhibition in the absence of MAGL leads to attenuation of melanoma tumor growth. This study not only sheds light on the metabolic flexibility of melanomas but also suggests the exploration of targeted therapeutic interventions involving CES1 inhibition.

## Materials and methods

### Substances and reagents

JZL184 was purchased from MedChem Express (HY-15249); WWL113 (SML1179) and MAFP (M2939) were purchased from Sigma-Aldrich; URB597 (10,046) and THL (orlistat, 10,005,426) were purchased from Cayman chemical; all at HPLC purity of > 98%. ActivX™ TAMRA-FP serine hydrolase probe was purchased from Thermo-Scientific (88,318). All amino acids and metabolites and their deuterated standards were analytical grade and were purchased from Sigma-Aldrich (Switzerland). Antibodies and other reagents are described in their corresponding sections. Radiolabeled 2-oleoylglycerol ([^3^H]−2-OG) was obtained from the American Radiolabeled Chemicals, Inc., MO, USA (ART 0400–250 µCi).

### Animal experiments

C57BL/6 J *BRaf*^*CA*^*, Pten*^*loxP/loxP*^*, and Pten*^*loxP/loxP*^*, Tyr::CreER*^*T2*^ mice were imported from McMahon’s laboratory and bred to get the *Braf*^*CA*^*, Pten*^*loxP*^*, Tyr::CreER*^*T2*^ experimental mice. Animals were kept in a temperature-controlled and humidity-controlled room (23 ± 2ºC; 50 ± 1%) with a 12:12 h light–dark cycle, having access to food and water ad libitum. Animals were handled according to Swiss federal legislation and guidelines of the local animal facility at the University of Bern. The protocols were approved by the respective government authorities (Veterinäramt Kanton Bern, experimental license BE-79/19).

### Melanoma induction

A cohort of 24 male and female mice was randomly divided into two groups to prevent bias and melanoma tumors were induced at the age of 6 weeks: mice were anesthetized by intraperitoneal (i.p.) injection of a mix of Dorbene (0.5 mg/kg), Dormicum (5 mg/kg) and Fentanyl (0.05 mg/kg) in 0.9% sterile NaCl. The back of each mouse was shaved and depilated with “Veet” cream, and a spot was tattooed with Ketchum green tattoo paste (Ketchum, QKI147325087). After, 1 µl of 1 mg/ml 4-OH-Tamoxifen in DMSO was applied right beneath the tattooed spot. The liquid was left for 30 min and removed with a humid paper towel. The anesthesia was antagonized by subcutaneous injection of Alzan (2.5 mg/kg), Flumazenil (0.5 mg/kg), and Naloxone (1.2 mg/kg) in 0.9% sterile NaCl. This procedure was repeated for three consecutive days.

### *J*ZL184 treatment in vivo and sample collection

Six weeks post-induction, mice were administered daily for three weeks with 10 mg/kg of JZL184 or vehicle (Ethanol: Kolliphor RH 40: 0.9% NaCl (1:1:8)) by intraperitoneal (i.p.) injection. Tumor volume was calculated using the formula *area x height*, where tumor height was measured weekly using a digital caliper, and the tumor area was calculated using ImageJ 1.53q software [46]. At the end of the 3rd week of treatment, animals were sacrificed by i.p. injection of ketamine/xylazine mix (87.5/12.5 mg/kg), tissues were collected, snap-frozen, and stored at −80 °C. The samples from the non-treated group, which were used for LC–ESI–MS/MS analysis, were collected 12 weeks post-induction.

### Cell culture

HEK293 cells (human embryonic kidney; RRID:CVCL_0045) were purchased from ATCC—Global Bioresource Center (CRL-1573). For maintenance, the cells were cultured at 37 °C with 5% CO_2_ humidified atmosphere in fully supplemented media: Dulbecco’s Modified Eagle Medium (DMEM) high glucose (Sigma-Aldrich, D6546), supplemented with 10% fetal bovine serum (FBS, Sigma-Aldrich).

### Transfection and selection

The *CES1*-expressing vector pcDNA3.1( +)-P2A-eGFP was purchased at Sino Biological (OHu18153C). For the stable transfection, HEK293 cells were seeded into 6-well plates at 40%—60% confluency, allowing them to attach for 24 h in fully supplemented media. Then, the cells were transfected with 2 µg of plasmid using jetPRIME transfecting reagent (Polyplus, 114–07), following the manufacturer’s protocol and replacing the media with fresh media 4 h post-transfection. Two days post-transfection, cells were subjected to antibiotic selection with 400 µg/ml Geneticin (G418 Sulfate; Thermo Fisher, 10,131,027) for 2–3 weeks. To select the single-clone-derived colonies, the clonal selection was performed by seeding a single cell suspension in 96-well plates, followed by their expansion. The presence of *CES1* expression was confirmed by the reporter GFP signal, ABPP, and Western blot.

### 2-Oleoylglycerol hydrolysis assay

2-Oleoylglycerol (2-OG) hydrolysis assay was performed on the stable HEK293-CES1 cells. Cells were seeded in 24-well plates, 150.000 cells/well. 24 h post-seeding, cells were pre-incubated with 1 µM of JZL184, WWL113, or DMSO for 30 min. Then, 1 µM of 2-OG mix (1000:1 ratio of 2-OG to [^3^H]−2-OG) was added for 30 min. To stop the reaction the cells and the supernatant were mixed with ice-cold methanol/chloroform (1:1, v/v, 1 mL). The samples were vortexed thoroughly and centrifuged at 16,000 × g, 10 min, 4 °C. The aqueous and organic phases were collected in scintillation vials, mixed with 3 ml of scintillation cocktail Ultima Gold (Revvity Health Sciences, Inc., 60,133,299), and analyzed on Packard Tri-Carb 2100 TR scintillation counter. The normalized counts are defined as % of hydrolyzed 2-OG relative to DMSO control.

### Mouse tissue sample preparation

The tissues were cryopulverized with the use of a mortar and pestle. After, the samples were homogenized by bead beating with chrome steel 2.3 mm beads (BioSpec, NC0605481), using a Mini-Beadbeater-24 (Bio Spec Products Inc.) for 1 min in 2 mg/ml solution of collagenase IV (Worthington, LS004189) in PBS + CaCl^2+^ + Mg^2+^. The tumor samples were incubated at 37 °C, shaking for 2 h, and the skin samples for 3 h. After, the samples were filtered with 70 µm filters and centrifuged at 16.000 rpm, 4 °C for 25 min. The supernatant was transferred to a new tube, and the centrifugation was repeated. This yielded the membranal fraction as a pellet and the cytosolic fraction as the supernatant. Total protein was quantified by Pierce™ BCA Protein Assay Kit (Thermo-Scientific, 23,225) following the manufacturer’s protocol. The samples were diluted to 3 mg/ml and kept at −80 °C.

### Activity-based protein profiling (ABPP)

ABPP experiments were performed using cytosolic protein fraction preparations obtained from mouse tumor and skin samples. To identify the serine hydrolases of interest, 30 µg of proteins were preincubated with indicated concentrations of MAFP, WWL113, JZL184, URB597, THL, or DMSO as control for 30 min at room temperature (RT), shaking. Then, the samples were labeled with a TAMRA-FP serine hydrolase probe at a final concentration of 125 nM for 30 min at RT. The reaction was stopped with 4 × Laemmli Buffer (200 mM Tris–HCl, pH 6.8, 8% (w/v) sodium dodecyl sulfate (SDS), 0.15% bromophenol blue, 40% glycerol, 5% (v/v) beta-mercaptoethanol), the samples were boiled at 70 °C for 5 min, cooled down to RT and separated by SDS-PAGE. The gels were scanned with Typhoon 9400 imager using TAMRA filter (λ_ex_ 550 nm and λ_em_ 570 nm). After, the gels were stained with coomassie to quantify the homogeneity of sample loading or instead continued with the Western blot analysis. The ABPP experiments were also performed to confirm *CES1* expression. 20 µg of cell homogenates were used and the ABPP followed the same protocol described above.

### Western blot analysis

Protein extracts of the cytosolic fractions were separated using 10–12% SDS-PAGE, and the proteins were transferred onto a nitrocellulose membrane in transfer buffer (18 mM Tris, 140 mM glycine; 20% v/v methanol, pH 8.0) using the Trans-Blot Turbo Transfer System (BioRad, **#**1,704,150). The membranes were blocked with 5% non-fatty milk in tris-buffered saline with 0.1% Tween 20 (TBST) overnight at 4 °C and then incubated with corresponding primary antibodies for 2 h at RT. Afterward, the membranes were washed with TBST three times and incubated with the corresponding secondary antibody for 2 h at RT. Membranes were washed with TBST, and the presence of antibodies was detected with Typhoon FLA 9500 (GE Healthcare). The following antibodies were used for immunoblotting analysis: Rabbit anti-CES1 (ABclonal Cat# A11478, RRID:AB_2861576) primary antibody (1:3000), Alexa Fluor 488 Goat Anti-Rabbit (Thermo Fisher Scientific Cat# A-11034, RRID:AB_2576217) secondary antibody (1:3000). Rabbit anti-GAPDH (Cell Signaling Technology Cat# 2118, RRID:AB_561053) was used as a loading control (1:3000).

### Deglycosylation via PNGase F treatment

Cytosolic fraction of mouse melanoma and skin tissues (20 μg) were deglycosylated by treatment with PNGase F at 37 °C for 17 h. The samples were labeled with TAMRA-FP serine hydrolase probe (125 nM final concentration) for 30 min and separated by SDS-PAGE.

### Sample preparation for LC–ESI–MS/MS assay

Mouse melanoma and skin samples were cryopulverized with the use of a mortar and pestle and transferred to 2 ml XXTuff reinforced microvials (Bio Spec, 330TX), weighted, and 4 volumes of acetonitrile (for endocannabinoids and bioactive lipids) or 1 mM EDTA in H_2_O (for amino acids and neurotransmitters) were added. The homogenization was performed by bead beating with chrome steel 2.3 mm beads (BioSpec, NC0605481) using a Mini-Beadbeater-24 (Bio Spec Products Inc.). For protein precipitation, amino acids, and neurotransmitters analysis, 50 μl of the sample were mixed with 200 μl of methanol (MeOH) and centrifuged at 16.000 rpm, 4 °C for 10 min. For endocannabinoids and bioactive lipids analysis, this step was avoided. Then, 50 μl of supernatant was recovered, mixed with 5 μl of internal standard, and 10 μl were injected into the LC–ESI–MS/MS system.

### LC–ESI–MS/MS method for amino acid and neurotransmitter quantification

A hybrid triple quadrupole 4000 QTRAP mass spectrometer (AB Sciex Concord, Ontario, Canada) coupled to a Shimadzu UFLC (Shimadzu Corporation, Kyoto, Japan) with a cooled auto-sampler was used. Sample temperature was maintained at 4 °C in the auto-sampler during analysis. The chromatographic separation was performed on the LC column Imtakt Intrada Amino acid WAA34, 100 × 3 mm; 3 µm, maintained at 35 °C. The mobile phase was composed of 100 mM ammonium formate in water (Mobile Phase A) and acetonitrile: water: formic acid (95:5:0.3, v/v/v) (Mobile Phase B). The system was operated in positive mode. Peaks were integrated, and the Analyst software version 1.6.3 (AB Sciex Concord, Ontario, Canada) was used for quantification. Identification of analytes in samples was based on precursor and product ion *m/z* values and LC retention times with standards in a multiple reaction monitoring (MRM) method. Further details regarding LC–ESI–MS/MS conditions are described in Supplementary Material.

### LC–ESI–MS/MS method for endocannabinoids and bioactive lipids quantification

A hybrid triple quadrupole 4000 QTRAP mass spectrometer (AB Sciex Concord, Ontario, Canada) coupled to a Shimadzu UFLC (Shimadzu Corporation, Kyoto, Japan) with a cooled auto-sampler was used. Sample temperature was maintained at 4 °C in the auto-sampler during analysis. The chromatographic separation was carried out on the Reprosil‐PUR C18 column, 3 μm particle size; 2 × 50 mm, Dr. A. Maisch, High-Performance LC‐GMBH (Ammerbuch, Germany) maintained at 40 °C. The system was operated in positive and negative modes with two different elution mobile phases and gradient methods. In positive mode, the elution mobile phases were composed of 2 mM ammonium acetate: water: formic acid (95:5:0.1, v/v/v) (Mobile Phase A) and 2 mM ammonium acetate in methanol (Mobile Phase B). The gradient started at 15% B, increasing linearly to 70% at 3.5 min, then to 99% at 8 min, maintained until 12 min, with subsequent re‐equilibration at 15% for 2.5 min. The flow rate was 0.35 ml/min. In negative mode, the organic mobile phase (B) was substituted with acetonitrile 0.1% formic acid to minimize crosstalk between analytes with the same mass ([M‐H]‐, m/z 351 PGE2/D2). The gradient started at 5% B, increasing linearly to 40% at 3 min, then to 65% at 9 min, and linearly again to 95% at 10 min; this was maintained until 14 min, with subsequent re‐equilibration at 5% for 3 min. The flow rate was 0.3 ml/min. Peaks were integrated, and the Analyst software version 1.6.3. (AB Sciex Concord, Ontario, Canada) was used for quantification. Identification of compounds in samples was confirmed by comparison of precursor and product ion *m/z* values and LC retention times with standards. Further details regarding LC–ESI–MS/MS conditions are described in Supplementary Material.

### Samples biotinylation

Two hundred µl of each sample (3 mg/ml) were mixed with ActivX™ Desthiobiotin-FP Serine Hydrolase Probe (Thermo-Scientific, 88,317) at a final concentration of 2 μM. The samples were vortexed and incubated at RT, shaking for 1 h. The excess of the probe was washed with PBS using Vivaspin**®** 2, 2.000 MWCO Hydrosart filtration columns (Sartorius, VS02H91), centrifuged at 5.000 g, 4 °C for 20 min. The supernatant was collected in a new tube and diluted with 2.5% SDS until the final concentration of 0.5% SDS. The samples were incubated at 95 °C for 5 min to denature the proteins. After cooling down to RT, the samples were diluted with PBS until a final concentration of 0.2% SDS and incubated with Pierce™ Avidin Agarose beads (Thermo-Scientific, 20,219) for 1 h while rotating. The beads were isolated by centrifugation at 1.600 g for 1 min and washed with 0.2% SDS (3 times) and PBS (5 times) using Micro Bio-Spin™ Columns (BioRad, 7,326,204). The beads were then transferred to 1.5 ml screw-cap tubes and labeled for submission.

### Proteomics. Sample preparation and LC–ESI–MS/MS analysis

The proteomic analysis was carried out at the Protein and Proteomics Analyses Functional Genomics Centre, Zurich. The beads were washed twice with 100 μl of 10 mM Tris, 2 mM CaCl_2_, pH 8.2, and the wash was discarded; next, the reduction and alkylation with TCEP and chloroacetamide were performed with 45 μl of 10 mM Tris, 2 mM CaCl2, pH 8.2 buffer and 5 μl of Trypsin (100 ng/μl in 10 mM HCl). The digestion was performed at 800 rpm, 37 °C for 4 h. Supernatants were collected, and the peptides were extracted from beads with 150 μl of 0.1% TFA 50% acetonitrile solution. The supernatants were combined and dried. The digested samples were dried and dissolved in 20 μl 0.1% formic acid and transferred to the autosampler vials for LC–ESI–MS/MS analysis. 2 μl were injected on a nanoAcquity UPLC coupled to a Q-Exactive mass spectrometer (Thermo-Scientific).

### Statistical analyses

The acquired MS data from the proteomics analysis were converted to a Mascot Generic File format (.mgf files) and were processed for identification using the Mascot search engine (Matrix Science). The spectra were searched against the *Mus musculus* database. The following modifications were set—variable modifications: Oxidation (M); fixed modifications: carbamidomethylation ©. The differential expression analysis was performed by the Functional Genomics Center, Zurich. The report is stored in the LIMS system *bfabric* [47] under order 31,647, with the work unit name 288,771. The protein identification and quantification were performed using FragPipe. For filtering and normalizing the data, generating visualizations, and computing differential expression analysis, R statistical software package *prolfqua* was used [48]. To further improve the power of the differential expression test, the protein variances were moderated [49]. Gel-based ABPP band intensity was quantified using ImageJ 1.53q software, and data are shown as the mean ± standard deviation (SD). LC–ESI–MS/MS data was analyzed by two-tailed paired t-test; the multiple comparisons were analyzed by two-way analysis of variance (ANOVA) and Tukey’s or Šídák’s post hoc test, where applicable. The post hoc test was only performed when *F* in ANOVA achieved *P* < 0.05. To analyze potential associations between the analytes in skin and melanoma tissues, Spearman correlation coefficients were calculated (two-tailed, 95% confidence interval). All statistical analyses were performed using GraphPad Prism for Microsoft Windows® (Versions 9, GraphPad software®, CA, USA) where *P* < 0.05 was accepted as significant; *P < 0.05, **P < 0.01, ***P < 0.001, ****P < 0.0001.

## Results

### Differential regulation of bioactive lipids in melanoma

**Fig. 3:**
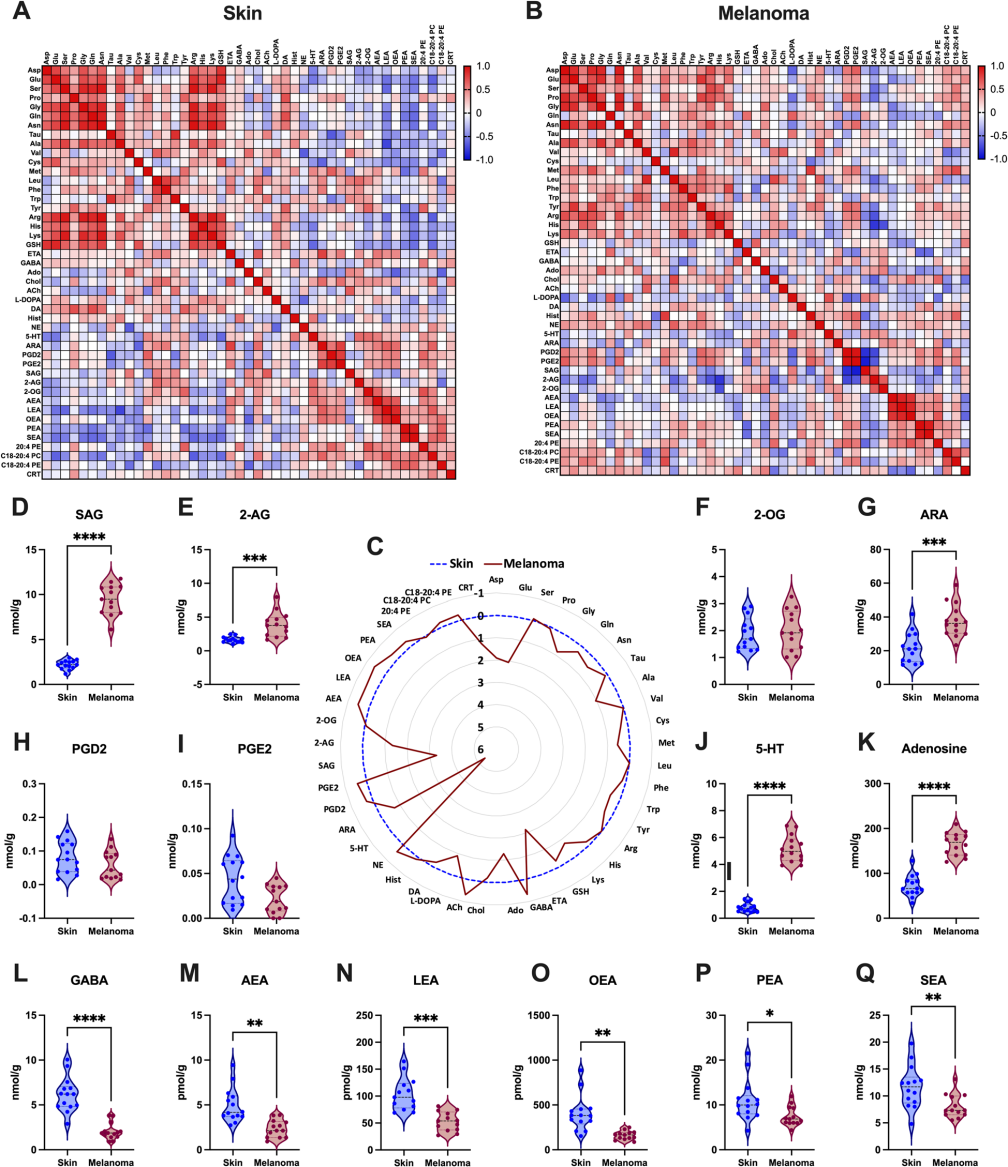
Metabolic signatures reveal that SAG, 2-AG, and ARA are increased in melanoma. Spearman correlation heatmaps of quantified metabolites in (**A**) skin and (**B**) melanoma. Red color indicates positive (> 0), white color indicates zero (0), blue color indicates negative (< 0) correlation, and the color intensity represents variation in the levels across the color scale. **C** Radar chart illustrating the levels of amino acids, neurotransmitters, and bioactive lipids in melanoma and normal skin, quantified by LC–ESI–MS/MS. Data are shown as fold change of the mean values, melanoma levels shown in red, and skin levels in blue, using skin levels as the baseline (fold change 0). **D**-**Q** Representative charts of endogenous levels of bioactive lipids in melanoma and skin. Data are shown as violin plots (*N* = 14 for each group) and analyzed by two-tailed paired t-test; ***P < 0.001; ****P < 0.0001. Abbreviations: 2-AG, 2-arachidonoylglycerol; 2-OG, 2-oleoylglycerol; 20:4 PE, 1,2-diarachidonoyl-sn-glycero-3-phosphoethanolamine; 5-HT, serotonin; Ach, acetylcholine; Ado, L-adenosine; AEA, anandamide; Ala, L-alanine; ARA, arachidonic acid; Arg, L-arginine; Asn, L-asparagine; Asp, L-aspartic acid; C18-20:4 PC, 1-(1Z-octadecenyl)−2-arachidonoyl-sn-glycero-3-phosphocholine; C18-20:4 PE, 1-(1Z-octadecenyl)−2-arachidonoyl-sn-glycero-3-phosphoethanolamine; Cho, choline; CRT, corticosterone; Cys, L-cysteine; ETA, ethanolamine; GABA, gamma-aminobutyric acid; Glu, L-glutamic acid; Gln, L-glutamine; Gly, L-glycine; GSH, glutathione; His, L-histidine; Hist, histamine; LEA, linoleoyl ethanolamide; Leu, L-leucine; Lys, L-lysine; Met, L-methionine; NE, norepinephrine; OEA, oleoyl ethanolamide; PEA, palmitoyl ethanolamide; PGD2, prostaglandin D2; PGE2, prostaglandin E2; Phe, L-phenylalanine; Pro, L- proline; SAG, 1-stearoyl-2-arachidonoyl-sn-glycerol; SEA, stearoyl ethanolamide; Ser, L-serine; Tau, L-taurine; Trp, L-tryptophan; Tyr, L-tyrosine; Val, L-valine

To assess the metabolic profiles of malignant melanoma and normal skin tissues, we employed LC–ESI–MS/MS-based targeted metabolomics on samples from skin and melanoma tissues obtained from the induced *B6-Tyr::CreER*^*T2*^*; BRaf*^*CA*^*; Pten*^*loxP*^ mice (Fig. 3). We first investigated potential changes in the metabolic signatures obtained by targeted metabolomicsTo further investigate the metabolic differences between malignant melanoma and normal skin tissues, we compared their metabolic profiles using a radar chart, emphasizing key variations in metabolite abundances (Fig. 3C). The most striking difference was observed in serotonin (5-HT) levels, which showed a 5.3-fold increase in melanoma tissues (Fig. 3J). Given our interest in the 2-AG-arachidonate-prostaglandin axis, another notable change observed in melanoma tissues was in the increased levels of SAG 3.3-fold (Fig. 3D). Additionally, 2-AG and ARA levels were increased in melanoma by 1.3- and 0.7-fold, respectively (Fig. 3E,G). This suggested a potential increase in the enzymatic activity responsible for the hydrolysis of SAG to 2-AG and, subsequently, from 2-AG to ARA. Nevertheless, the levels of prostaglandins, downstream products of ARA metabolism (Fig. 1), remained unchanged between melanoma and normal skin tissues (Fig. 3H,I). Furthermore, we observed a significant increase in other bioactive molecules, such as adenosine (Fig. 3K) and GABA (Fig. 3L). In contrast, all long-chain fatty acid *N*-acylethanolamines—AEA, LEA, PEA, OEA, SEA—were significantly reduced in melanoma compared to normal skin (Fig. 3M-Q). Supplementary Figure S1 shows additional analyzed bioactive lipids and neurotransmitters.

### Expression of Ces1 in melanoma and normal skin

Because of the increased SAG, 2-AG, and ARA levels in melanoma tissues and our interest in the source of ARA, we conducted a targeted proteomics analysis to identify the enzymes responsible for the hydrolysis of these bioactive lipids. It is important to note that the desthiobiotin-FP serine hydrolase probe used in this study does not detect DAGLα/β, the enzymes responsible for SAG hydrolysis. Therefore, our investigation centered on serine hydrolases capable of hydrolyzing 2-AG and leading to ARA production.

**Fig. 4:**
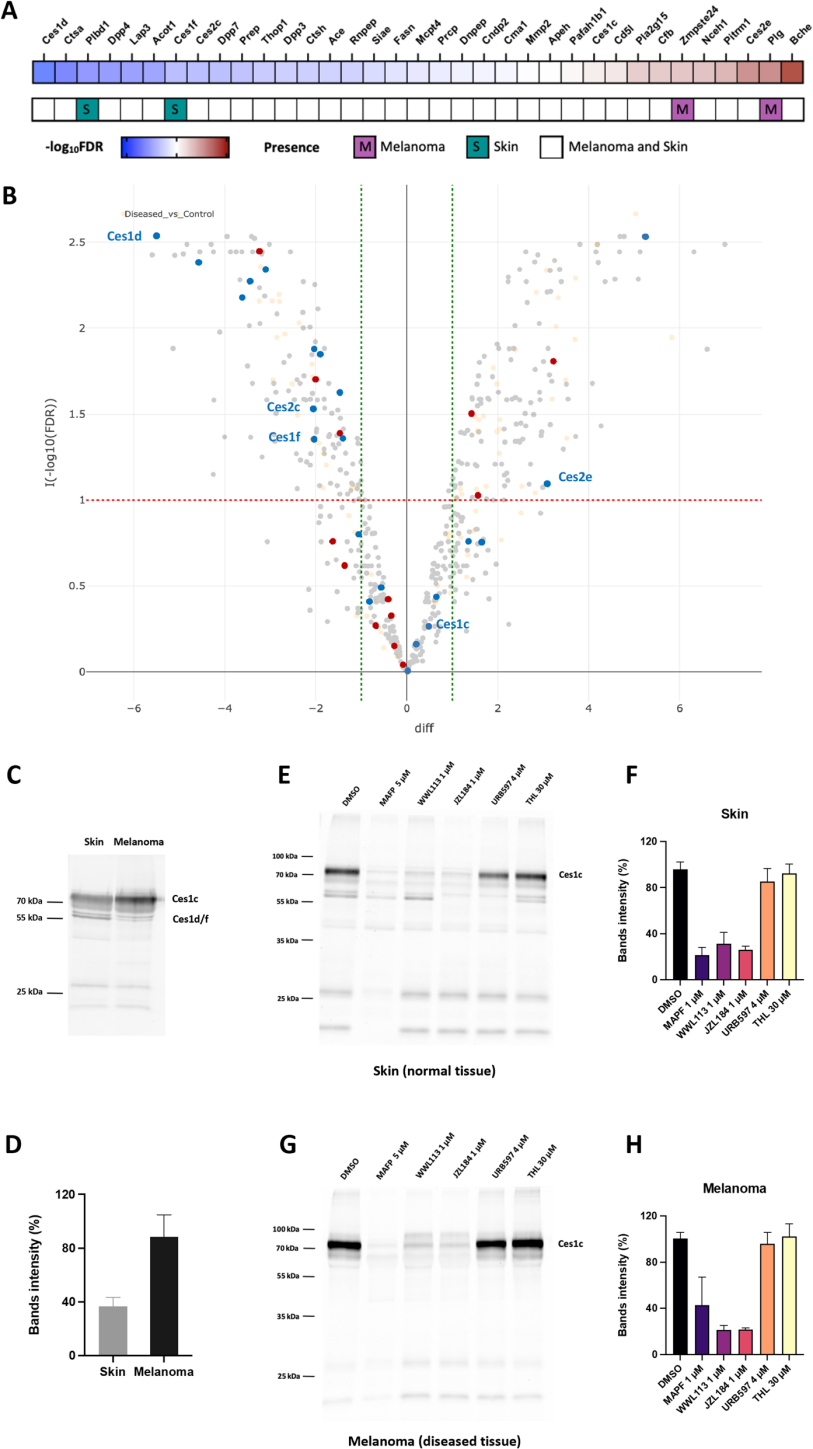
Serine hydrolases found in melanoma and normal skin tissues. **A** Heatmap depicting the − log_10_ transformed False Discovery Rate (FDR) of the differential expression analysis of serine hydrolases between mouse melanoma and skin. Red color indicates positive (> 0), white color indicates zero (0), blue color indicates negative (< 0) expression differences and the color intensity represents variation in the levels across the color scale. Pink color represents proteins present only in melanoma; green color represents proteins present only in the skin. **B** Volcano plot showing − log_10_ transformed FDR of the differential analysis between Diseased (melanoma) and Control (skin) groups. Red line indicates the − log_10_(FDR) of FDR = 0.1, green lines indicate the difference of −1 and + 1. Differences and FDRs estimated using missing value imputation are shown in orange. Metabolic serine hydrolases are shown in blue; serine proteases are shown in red. Isoforms of Ces1 and Ces2 are indicated on the graph. **C** Representative gel-based ABPP of mouse melanoma and skin samples. Samples were incubated with the activity probe TAMRA-FP (125 nM) for 30 min at RT. **D** Quantification of Ces1c band intensity from the gel-based ABPP. Data are shown as mean ± SD from three independent experiments. Representative gel-based ABPP of mouse skin (**E**) and melanoma (**G**) samples. Samples were incubated with the indicated concentrations of MAPF, WWL113, JZL184, URB297, and THL for 30 min at RT before incubation with the activity probe TAMRA-FP (125 nM) for 30 min at RT. Quantification of Ces1c band intensity from the gel-based ABPP of mouse skin (**F**) and melanoma (**H**) samples. Data are shown as mean ± SD from four independent experiments, performed in triplicates. Abbreviations: M – melanoma, S – skin

Our chemoproteomics analysis yielded 35 serine hydrolases, comprising 22 metabolic serine hydrolases and 13 serine proteases (Fig. 4). Notably, two serine proteases—plasminogen (Plg) and the CAAX prenyl protease 1 homolog (Zmpste24)—were detected only in melanoma, while two metabolic serine hydrolases—phospholipase B-like 1 (Plbd1) and carboxylesterase 1F (Ces1f)—were found only in normal skin. The remaining 31 enzymes were detected in both melanoma and normal skin tissues (Fig. 4A).

Unexpectedly, the most frequently expressed 2-AG-hydrolyzing enzymes MAGL, ABHD6, and ABHD12 were absent in melanoma and skin tissues. The only identified enzymes known to hydrolyze 2-AG were the carboxylesterases Ces1 and Ces2. Three Ces1 isoforms were detected, Ces1c, Ces1d, and Ces1f, along with two Ces2 isoforms, Ces2c and Ces2e (Fig. 4A,B). Another Ces2 isoform involved in 2-AG hydrolysis, Ces2g, was not detected in melanoma or skin tissues. Interestingly, differential expression analysis showed that Ces1c expression did not significantly differ between melanoma and normal skin. In contrast, Ces1d expression was dramatically reduced in melanoma, and Ces1f was only detected in the normal skin (Fig. 4A,B). The expression profiles of Ces2 isoforms also varied, with Ces2e being elevated in melanoma, while Ces2c was more abundant in normal skin (Fig. 4A,B).

Activity-based protein profiling (ABPP) of melanoma and skin samples revealed a very prominent band around ∼70 kDa (Fig. 4C). This band was more pronounced in melanoma samples, suggesting an increased enzyme activity compared to the normal skin (Fig. 4D). In addition to MAFP, a potent non-specific inhibitor of serine hydrolases, CES1 was also inhibited by the MAGL inhibitor JZL184 and WWL113, known CES1/Ces1 inhibitors in both humans and mice [20, 50, 51] (Fig. 4E-H). CES1 was identified as the Ces1c isoform, previously described to appear at ∼70 kDa due to extensive glycosylation, thus distinguishing it from other Ces1 isoforms (Supplementary Figure S2) [52].

### Ces1 inhibition impairs melanoma growth in vivo

**Fig. 5:**
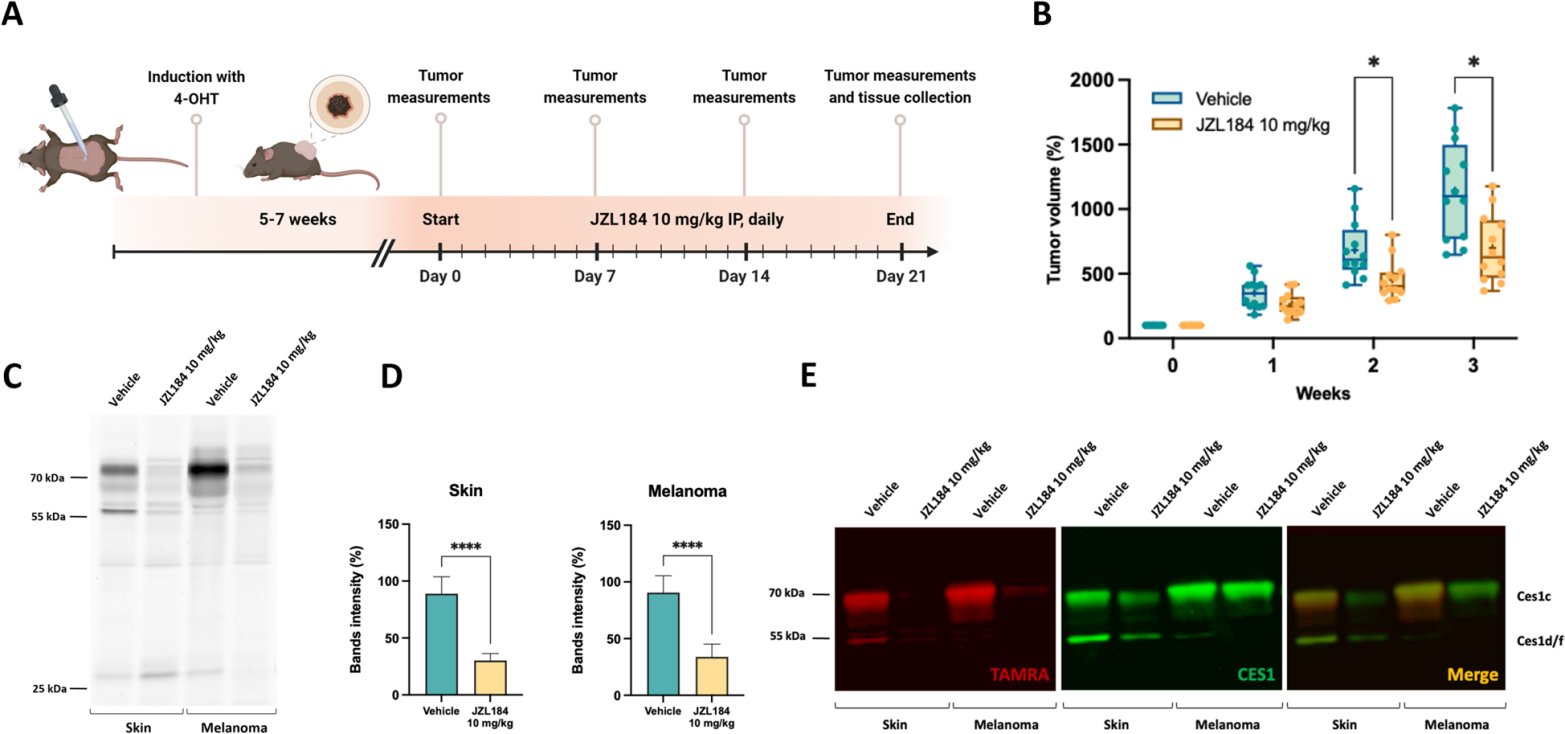
CES1 inhibition impairs melanoma growth. **A** Schematic representation of the experimental design and treatment schedule. **B** Normalized tumor volume of JZL184- and vehicle-treated groups. The initial tumor volume was considered 100% for both groups. JZL184 and vehicle (Ethanol: Kolliphor RH 40: 0.9% NaCl (1:1:8)) were administered by i.p. injection. Data are shown as mean ( +) and minimum to maximum whiskers (*N* = 14 for each group) and analyzed by two-way ANOVA with multiple comparisons and Šídák’s post hoc test; *P < 0.05. **C** Representative gel-based ABPP of mouse melanoma and skin samples after the in vivo treatment with JZL184 or vehicle. Samples were incubated with the activity probe TAMRA-FP (125 nM) for 30 min at RT. **D**) Quantification of the Ces1c band intensity from the gel-based ABPP of mouse melanoma and skin samples after the in vivo treatment with JZL184 or vehicle. Data are shown as mean ± SD from two independent experiments (*N* = 12 for each group) and analyzed by unpaired t-test ****P < 0.0001. **E** Representative gel-based ABPP and immunoblot overlay of mouse melanoma and skin samples after the in vivo treatment with JZL184 or vehicle. Samples were first incubated with the activity probe TAMRA-FP (red) (125 nM) for 30 min at RT, then transferred to a nitrocellulose membrane and blotted with anti-CES1 antibody (green)

Given the elevated levels of SAG, 2-AG, and ARA observed in mouse melanomas, along with the detection of active Ces1 isoforms in both melanoma and skin tissues, we next evaluated the effects of in vivo Ces1 inhibition on melanoma progression. Based on the in vitro results (Fig. 4E-H) and the absence of MAGL (Fig. 4A), we selected JZL184 for our in vivo study, as this MAGL-specific inhibitor also covalently inhibits CES1 [20, 50, 51] and the compound is established as for in vivo studies. Mice were treated with 10 mg/kg of JZL184 or vehicle, administered daily via intraperitoneal injection (i.p.) for three consecutive weeks (Fig. 5A). After two weeks of daily treatment, JZL184 significantly but moderately slowed melanoma progression compared to the vehicle-treated group, an effect that persisted through the third week of treatment (Fig. 5B). Since this melanoma model is very aggressive, this effect was also therapeutically significant.

To confirm the efficacy of the in vivo Ces1 inhibition, we conducted ex vivo ABPP on both melanoma and skin samples, showing that Ces1 was effectively inhibited in the JZL184-treated group, indicated by a clear reduction in the ex vivo ABPP(Fig. 5C,D). The ABPP/WB overlay further validated the in vivo inhibition of the Ces1c isoform, confirming it as the primary target of JZL184 and reinforcing the specificity of the compound in inhibiting Ces1 activity in the treated samples (Fig. 5E). Notably, inhibition of Ces1d/f isoforms was also observed in skin samples (Fig. 5E), demonstrating that JZL184 effectively targets multiple Ces1 isoforms in vivo.

### Covalent inhibition of Ces1 leads to increased 2-AG and 2-OG skin levels in vivo

**Fig. 6:**
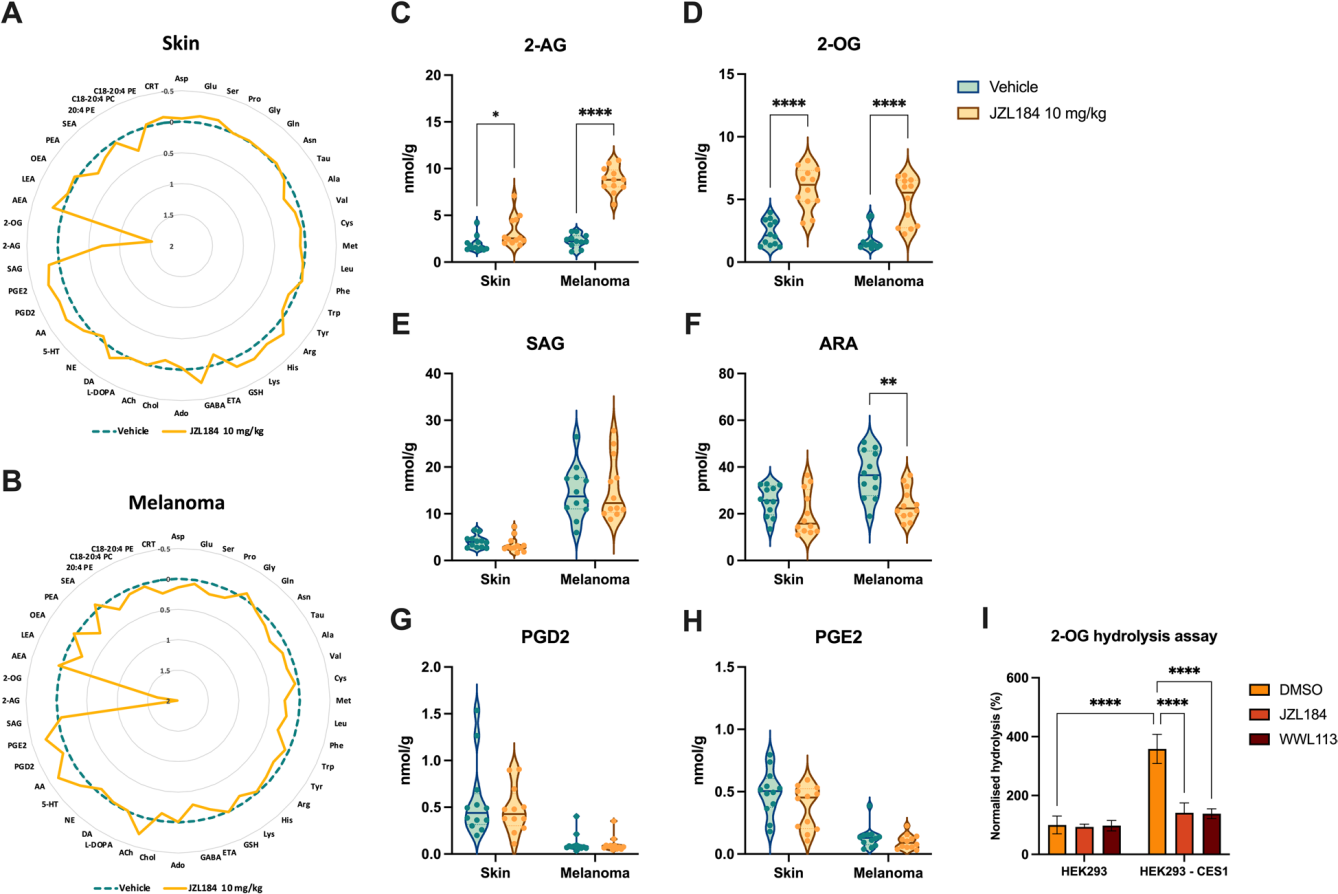
JZL184 increases 2-AG and 2-OG levels in vivo. Radar charts illustrating the levels of amino acids, neurotransmitters, and bioactive lipids in (**A**) skin and (**B**) melanoma samples, quantified by LC–ESI–MS/MS. Data are shown as mean values of bioactive molecule amounts of the JZL184-treated group (yellow) compared to the vehicle group (green) used as 100%. **C**-**H** Representative charts of endogenous levels of bioactive lipids in melanoma and skin after JZL184 treatment in vivo. Data are shown as violin plots (*N* = 12 for each group) and analyzed by two-way ANOVA with multiple comparisons and Tukey’s post hoc test; *P < 0.05; **P < 0.01; ****P < 0.0001. **I** Normalized % of 2-OG hydrolysis in HEK293 and HEK293-CES1 cells. Samples were preincubated with 1 μM JZL184 or WWL113 for 30 min, followed by 1 μM 2-OG incubation for 30 min. Data are shown as mean ± SD from three independent experiments performed in triplicates and analyzed by two-way ANOVA with multiple comparisons and Šídák's post hoc test; ****P < 0.0001. Abbreviations: 2-AG, 2-arachidonoylglycerol; 2-OG, 2-oleoylglycerol; 20:4 PE, 1,2-diarachidonoyl-sn-glycero-3-phosphoethanolamine; Ach, acetylcholine; Ado, L-adenosine; AEA, anandamide; Ala, L-alanine; ARA, arachidonic acid; Arg, L-arginine; Asn, L-asparagine; Asp, L-aspartic acid; C18-20:4 PC, 1-(1Z-octadecenyl)−2-arachidonoyl-sn-glycero-3-phosphocholine; C18-20:4 PE, 1-(1Z-octadecenyl)−2-arachidonoyl-sn-glycero-3-phosphoethanolamine; Cho, choline; CRT, corticosterone; Cys, L-cysteine; ETA, ethanolamine; GABA, gamma-aminobutyric acid; Glu, L-glutamic acid; Gln, L-glutamine; Gly, L-glycine; GSH, glutathione; His, L-histidine; LEA, linoleoyl ethanolamide; Leu, L-leucine; Lys, L-lysine; Met, L-methionine; NE, norepinephrine; OEA, oleoyl ethanolamide; PEA, palmitoyl ethanolamide; PGD2, prostaglandin D2; PGE2, prostaglandin E2; Phe, L-phenylalanine; Pro, L- proline; SAG, 1-stearoyl-2-arachidonoyl-sn-glycerol; SEA, stearoyl ethanolamide; Ser, L-serine; Tau, L-taurine; Trp, L-tryptophan; Tyr, L-tyrosine; Val, L-valine

We performed an in-depth targeted metabolomic analysis using LC–ESI–MS/MS to quantify the alterations in molecular signatures of the mouse melanomas and skin following in vivo administration of JZL184 (Fig. 6A,B). As expected, the most significant changes were observed in the levels of 2-AG and 2-OG, which strongly increased after in vivo JZL184 treatment. Specifically, 2-AG showed a twofold increase in melanoma and a 0.7-fold increase in the skin, while 2-OG exhibited a 1.7-fold increase in melanoma and a 1.5-fold increase in the skin (Fig. 6C,D). These findings indicate that Ces1 mediates the hydrolysis of both 2-AG and 2-OG in melanoma and normal skin tissues of the *B6-Tyr::CreER*^*T2*^*; BRaf*^*CA*^*; Pten*^*loxP*^ mice. To confirm the ability of CES1/Ces1 to hydrolyze 2-AG/2-OG, we generated a stable CES1-expressing cell line using HEK293 cells, which hydrolyzed approximately 3.5 times more 2-OG than the wild type cells, and this hydrolysis was effectively inhibited by the specific CES1 inhibitors JZL184 and WWL113 (Fig. 6I).

Another interesting observation was the 0.3-fold reduction in ARA levels in melanomas following in vivo JZL184 administration (Fig. 6F), whereas the levels of SAG and prostaglandins remained consistent between the two treatment groups (Fig. 6E,G,H). Additionally, melanomas from the JZL184-treated group exhibited elevated levels of GABA and phenylalanine (Supplementary Figure S3). The long-chain fatty acid *N*-acylethanolamines—AEA, LEA, PEA, OEA, SEA – did not show any variations after the treatment (Supplementary Figure S4).

#### Distinct expression patterns of CES1 and MAGL in melanoma from RNA-seq data

**Fig. 7:**
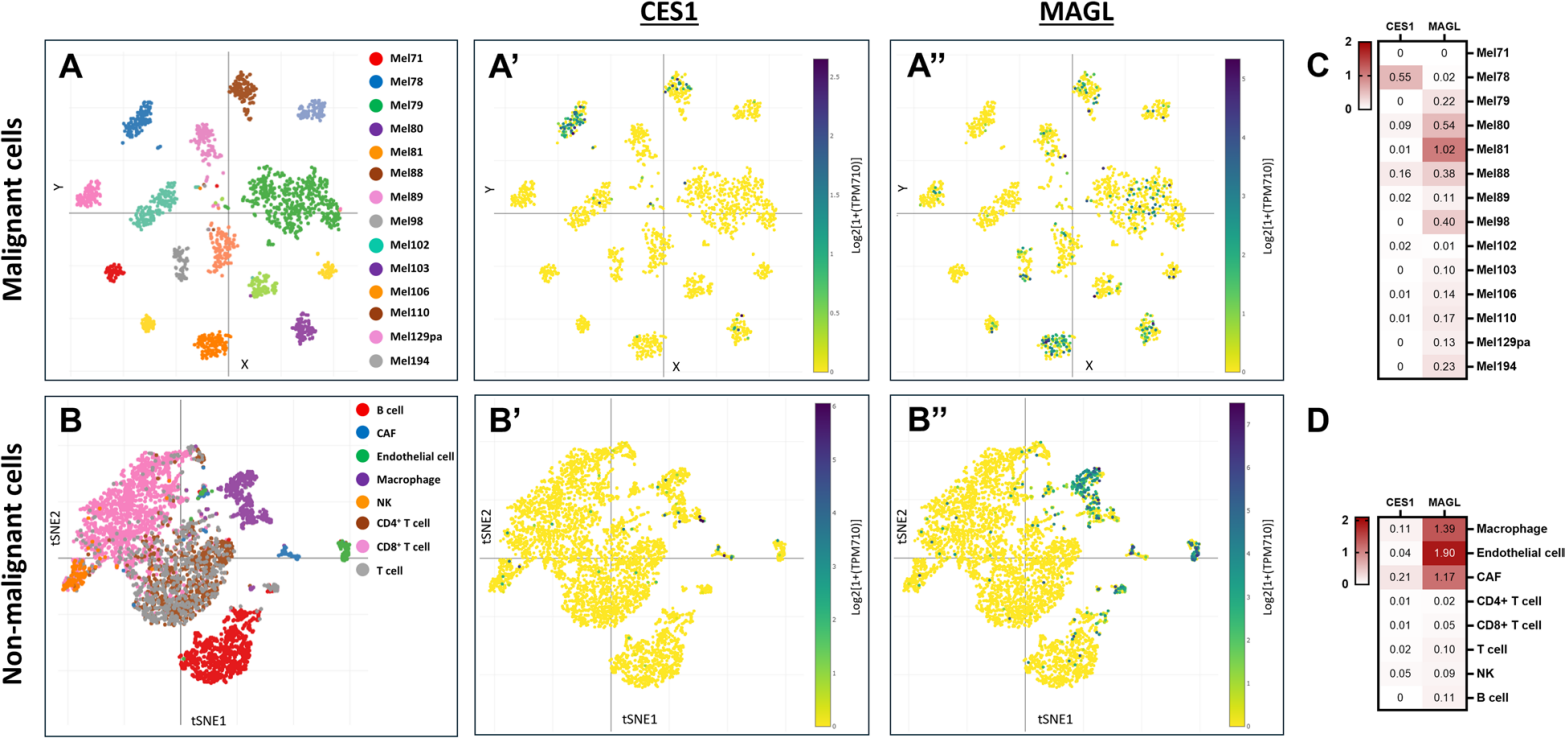
CES1 and MAGL expression in human malignant melanoma. Scatter plots of the integrative analysis of scRNA-seq data of human melanoma samples with a t-stochastic neighbor embedding (t-SNE) of single-cell profiles (dots) of (**A**) malignant and (**B**) non-malignant cells, colored by (**A**) tumor or by (**B**) post hoc annotation. **A** Only tumors with at least 50 malignant cells are shown. Expression of CES1 in (A’) malignant and (B’) non-malignant cells. Expression of MAGL in (A’’) malignant and (B’’) non-malignant cells. Heatmaps depicting CES1 and MAGL expression in (**C**) malignant and (**D**) non-malignant cells. Red color indicates positive expression (> 0), white color indicates zero (0), and the color intensity represents variation in the levels across the color scale. Abbreviations: CAF, cancer-associated fibroblast; NK, natural killer cell [53, 54]

Following up on our findings regarding the role of CES1/Ces1 in 2-AG/2-OG hydrolysis and melanoma progression, we analyzed the available RNA sequencing (RNA-seq) data on the expression of 2-AG-hydrolysing enzymes in both human malignant melanoma and human skin tissues. The expression of CES1 in human skin was confirmed by ABPP/WB overlay using normal human skin tissue (Supplementary Figure S5). Figure 7 shows an integrative analysis of single-cell RNA sequencing (scRNA-seq) data for CES1 and MAGL expression across 33 human melanoma tissues, as reported by Tirosh et al. and Jerby-Arnon et al. [53, 54]. This analysis is complemented by Supplementary Figure S6, which shows the expression levels of other 2-AG-hydrolyzing enzymes—ABHD6 and ABHD12.

The scRNA-seq data showed that CES1 expression in malignant melanoma cells is generally lower compared to MAGL (Fig. 7A’,A’’). Among the 2-AG-hydrolyzing enzymes, ABHD12 was the most widely expressed (Supplementary Figure S6A’’). Non-malignant immune cells exhibited low CES1 expression, which was not associated with any specific cell type (Fig. 7B’). In contrast, MAGL was highly expressed in macrophages (Fig. 7B’’), as well as ABHD12 (Supplementary Figure S6B’).

Notably, in the malignant cells of the Mel78 sample, we found a remarkable case where CES1 expression was dramatically high (Fig. 7A’,C), while MAGL expression was nearly absent (Fig. 7A’’,C). This finding is particularly meaningful as it mirrors what we observed in the *BRaf*^*V600E*^/*Pten*^*−/−*^ mice, where high CES1 expression coincided with the absence of MAGL (Fig. 4A). Therefore, the specific expression pattern of 2-AG-hydrolyzing enzymes suggests differential roles of CES1 and MAGL in different melanoma subtypes.

## Discussion

Despite significant advances in melanoma treatment, the prognosis for metastatic *BRAF*^*V600E*^-driven melanoma remains poor [55], highlighting the critical need for a deeper understanding of its underlying mechanisms. Among the numerous pathways implicated in melanoma progression, the ECS potentially plays a role in tumor development and growth [8, 56]. Beyond their critical role as neuromodulators [57], endocannabinoids are involved in numerous physiological processes, including cell proliferation, differentiation, apoptosis, and immune response modulation [58–60]. In agreement, a dysregulation of endocannabinoid metabolism and signaling has been linked to various pathologies, including cancer, where it contributes to immune evasion, tumor growth, and metastasis [56, 59, 61].

In our study, we focused on the lipid remodeling and endocannabinoid-prostaglandin axis, as well as differences in serine hydrolase expression in melanoma versus normal skin. Employing LC–ESI–MS/MS, we examined the bioactive lipid profiles in a genetically engineered *BRaf*^*V600E*^/*Pten*^*−/−*^ mouse model. In melanoma tissue, we observed consistently reduced NAEs, which may result from decreased NAPLD activity [62, 63], but elevated levels of SAG, 2-AG, and ARA (Fig. 3). The significant increase in SAG levels observed in melanomas suggests a potential role in activating the MAPK/ERK signaling pathway. While diacylglycerols (DAGs) are predominantly recognized for their activation of various protein kinase C (PKC) isoforms [64, 65], they also mediate the activation of other proteins, including RasGRPs [66, 67]. RAS guanyl-releasing protein 1 (RasGRP1) functions as a guanine nucleotide exchange factor, and its activation by the DAGs leads to the activation of the RAS small GTPase protein—a member of the MAPK/ERK signaling pathway—through the exchange of bound GDP for GTP [68]. The presence of RasGRP1 has been confirmed in keratinocytes derived from the skin, and overexpression of this protein has been linked to the promotion of skin tumor formation [69], likely mediated through direct RAS activation [70, 71]. It has previously been shown that basal levels of total diacylglycerol were elevated fivefold in B16 melanoma cells compared to levels found in quiescent or proliferating Mel-ab melanocytes and appear to arise largely from the breakdown of phosphatidylinositol phospholipids accompanied by a significant rise in phospholipase C activity [72]. The genetically engineered mouse model used in this study carries melanocyte-specific *BRaf*^*V600E*^ and *Pten*^*KO*^ that drive the hyperactivation of the MAPK/ERK signaling pathway. The elevated levels of SAG observed in the tumors might potentiate this signaling cascade through PKC activation and enhanced RasGRP1 activation, thereby ensuring persistent and amplified MAPK/ERK pathway activation, thus promoting melanoma progression.

The hydrolysis of membranal SAG by DAG lipases -alpha o -beta leads to the formation of one of the most abundant endocannabinoids, 2-AG, and the structurally similar 2-OG [73, 74]. In our study, 2-AG levels were significantly increased in melanoma tissues compared to normal skin (Fig. 3E). While 2-AG has been implicated in both pro- and anti-tumorigenic activities, the balance of its effects may be context-dependent, depending on receptor signaling or degradation by serine hydrolases feeding the arachidonate pathways. For example, elevated 2-AG levels have been correlated with enhanced glioma tumor growth [75]. In contrast, the inhibition of the primary 2-AG-hydrolyzing enzyme, MAGL, led to elevated 2-AG levels and has been shown to impair cell migration in aggressive breast, ovarian, and melanoma cancer cell lines [76]. Moreover, MAGL inhibition has also been shown to reduce colon carcinoma progression [77] and impair the growth of non-small cell lung cancer [15]. Additionally, JZL184-mediated MAGL inhibition induced an anti-hyperalgesic effect in a mouse model of mechanical hyperalgesia [78]. The hydrolysis of 2-AG leads to the formation of ARA, elevated levels of which are known to promote melanoma progression and tumor proliferation [58, 79, 80]. ARA has also been implicated in the development of other types of cancer, including colorectal cancer [81, 82].

The chronic in vivo administration of JZL184 to the *BRaf*^*V600E*^/*Pten*^*−/−*^ mice further increased 2-AG and 2-OG levels and decreased ARA levels in melanoma and normal skin (Fig. 6C,D,F), leading to attenuated tumor growth (Fig. 5B). Although JZL184 is a specific MAGL inhibitor, the observed effects were attributed to CES1 inhibition, as a particularly striking finding from our study was the absence of MAGL and any other canonical 2-AG-hydrolyzing enzymes, such as ABHD6 or ABHD12 (Fig. 4A,B). While CES1 has been previously described to hydrolyze 2-AG [20, 83], the role of this enzyme in the skin and malignant melanoma has remained completely unexplored until now. This study is the first to demonstrate that metabolic pathways involving elevated levels of SAG, 2-AG, and ARA may contribute to melanoma progression, with CES1 directly influencing lipid metabolism within the melanoma tumor microenvironment.

Importantly, available scRNA-seq data on human samples confirms that CES1 is predominantly expressed in the liver [24, 26, 84], with particularly high expression levels in hepatocytes, the primary cell type of the liver [85, 86]. Interestingly, in the skin, CES1 appears to be expressed in fibroblasts, where it shows substantial expression, as well as in endothelial cells, stromal cells, and melanocytes, although at lower levels [84, 87–92]. In immune cells, CES1 expression has been detected in monocytes/macrophages, dendritic cells, and NK cells [91–93]. Therefore, the metabolism of bioactive lipids, such as SAG, 2-AG, and ARA may be compartmentalized within the tumor microenvironment, with different cell types potentially contributing to the production and hydrolysis of specific lipids.

We identified the Ces1c isoform as the most active serine hydrolase in melanoma tissues from the *BRaf*^*V600E*^/*Pten*^*−/−*^ mice (Fig. 4C,D; Supplementary Figure S2). Although the Ces1d and Ces1f isoforms were also detected, their prevalence in normal skin suggests a minor role in tumor progression (Fig. 4A,B). While our targeted proteomics data also identified Ces2c and Ces2e, which were previously reported to hydrolyze 2-AG [35], the primary Ces2 isoform Ces1g, which is generally associated with 2-AG hydrolysis [94], was not detected in our analysis. This suggests that CES1, particularly the Ces1c isoform, is the dominant enzyme responsible for 2-AG degradation in melanoma of the *BRaf*^*V600E*^/*Pten*^*−/−*^ mouse model. Beyond 2-AG, CES1 has also been shown to hydrolyze prostaglandins [95]. However, we did not observe a decrease in PGE2/PGD2 levels following in vivo JZL184 treatment and CES1 inhibition (Fig. 6G,H), indicating that the Ces1c isoform is not involved in PGE2/PGD2 degradation in *BRaf*^*V600E*^/*Pten*^*−/−*^ mice. Likewise, the lower anandamide and overall NAEs levels observed in the melanoma was not changed by JZL184 treatment, indicating that the biosynthesis of NAEs via *N*-acyl phosphatidylethanolamine phospholipase D (NAPEPLD) rather than hydrolysis by CES1 could be involved [62, 63].

Our biochemical findings align with the scRNA-seq data reported by Tirosh et al. and Jerby-Arnon et al., which showed a unique expression pattern of CES1 in malignant melanoma cells [53, 54]. While CES1 expression was generally lower than MAGL (Fig. 7A’,A’’), the notable exception of a tumor exhibiting high CES1 expression with nearly absent MAGL expression (Fig. 7A’,C) suggests a compensatory mechanism for 2-AG hydrolysis, where CES1 assumes a primary 2-AG-hydrolyzing role in the absence of MAGL. This variability in enzyme expression reflects metabolic flexibility and adaptations of melanoma tumors that could be exploited therapeutically. Furthermore, in the melanoma patient samples analyzed, CES1 expression did not associate with any specific immune cell type (Fig. 7B’). This suggests that the presence of CES1 signal in melanoma tissue may originate from various cell types, supporting the previously described scRNA-seq data [91–93]. In contrast, MAGL expression is highly associated with macrophages (Fig. 7B’’), similar to ABHD12 expression (Supplementary Figure S6B’). Future studies will have to explore the long-term effects of CES1 inhibition and the mechanism of action of 2-AG and other lipids, their roles in metastasis, and their impact on overall survival in melanoma, potentially paving the way for novel therapeutic approaches in melanoma treatment.

## Conclusions

Targeted metabolomics revealed distinct metabolic signatures in skin and *BRaf*^*V600E*^ driven melanoma tissues, showing notable changes such as increased levels of serotonin, SAG, 2-AG, and ARA, alongside decreased levels of NAEs. Using chemoproteomics, we show the expression of multiple Ces1 isoforms—Ces1c, Ces1d, and Ces1f—in mouse melanoma and normal mouse and human (Ces1) skin. The Ces1c isoform was identified as the primary enzyme driving 2-AG degradation, apparently associated with lipid metabolism and melanoma progression. The potential translational relevance of our findings is supported by scRNA-seq data from Tirosh et al*.* and Jerby-Arnon et al., which reported a case with elevated CES1 expression in the absence of MAGL. We hypothesize a compensatory mechanism driven by SAG that sustains 2-AG synthesis and hydrolysis, leading to ARA production and favoring tumor growth, as 2-AG is known to slow tumor progression and ARA promotes it. Importantly, CES1 was not responsible for the significantly lower NAE levels in melanoma tissues. In vivo inhibition of Ces1c significantly increased levels of 2-AG and 2-OG without changing the extractable bulk amount of ARA. CES1 inhibition in the tumor tissues led to a marked slowdown in melanoma growth, suggesting a role of 2-AG and potentially local ARA metabolism that cannot be easily detected in the tumor tissue, independent of prostaglandins. CES1 expression could thus be a potential marker for classifying these aggressive melanoma subtypes. Overall, the identification of CES1 activity in melanoma provides new insights into the metabolic adaptations of these tumors and opens avenues for exploring diagnostic or therapeutic opportunities for melanoma characterization and treatment.

## Supplementary Information

Supplementary Material 1.

## Data Availability

Data is provided within the manuscript or supplementary information files.

## Abbreviations

2-AG: 2-Arachidonoylglycerol
2-OG: 2-Oleoylglycerol
AAs: Amino acids
ABHD6: Alpha/beta-hydrolase domain-containing protein 6
ABHD12: Alpha/beta-hydrolase domain-containing protein 12
ACh: Acetylcholine
Ado: Adenosine
AEA: Anandamide
Ala: Alanine
ARA: Arachidonic acid
Arg: Arginine
Asn: Asparagine
Asp: Aspartic acid
C18-20:4 PC: 1-(1Z-octadecyl)-2-arachidonoyl-sn-glycero-3-phosphocholine
C18-20:4 PE: 1-(1Z-octadecyl)-2-arachidonoyl-sn-glycero-3-phosphoethanolamine
20:4 PE: 1,2-Diarachidonoyl-sn-glycero-3-phosphoethanolamine
CES: Carboxylesterases
CES1: Carboxylesterase 1
CES2: Carboxylesterase 2
Chol: Choline
CM: Cutaneous melanoma
CRT: Corticosterone
COX1: Cyclooxygenase 1
COX2: Cyclooxygenase 2
Cys: Cysteine
DAGLα: Diacylglycerol lipase α
DAGL β: Diacylglycerol lipase β
ECS: Endocannabinoid system
ETA: Ethanolamine
GABA: *gamma*-Aminobutyric acid
Glu: Glutamic acid
Gln: Glutamine
Gly: Glycine
GSH: Glutathione
His: Histidine
Hist: Histamine
LAT1: Large neutral amino acid transporter 1
LAT2: Large neutral amino acid transporter 2
LC–ESI–MS/MS: Liquid chromatography coupled to tandem mass electron ionization electrospray spectrometry
LEA: Linoleoyl ethanolamide
Leu: Leucine
Lys: Lysine
MAGL: Monoacylglycerol lipase
MeOH: Methanol
Met: Methionine
NAEs: N-acylethanolamines
NAPE-PLD: N-acyl phosphatidylethanolamine phospholipase D
NE: Norepinephrine
OEA: Oleoyl ethanolamide
PEA: Palmitoyl ethanolamide
PGD2: Prostaglandin D2
PGE2: Prostaglandin E2
Phe: Phenylalanine
PKC: Protein kinase C
Pro: Proline
RasGRP1: RAS guanyl-releasing protein 1
RNA-seq: RNA sequencing
RT: Room temperature
SAG: 1-Stearoyl-2-arachidonoyl-sn-glycerol
scRNA-seq: Single-cell RNA sequencing
SEA: Stearoyl ethanolamide
Ser: Serine
Tau: Taurine
Thr: Threonine
Trp: Tryptophan
Tyr: Tyrosine
Val: Valine
WB: Western blot
