# Supplementary material for: Carboxylesterase 1-mediated endocannabinoid metabolism in skin: role in melanoma progression in BRafV600E/Pten−/− mice
Source: Cancer Metab. 2025 Feb 11;13:8. doi: 10.1186/s40170-025-00378-2 (PMC11817774; doi:10.1186/s40170-025-00378-2)
Supplement: Supplementary file 1 — Supplementary Material 1. [file 40170_2025_378_MOESM1_ESM.pdf]

Supplementary Material

Carboxylesterase 1-Mediated Endocannabinoid Metabolism in Skin: Role in Melanoma Progression in *BRaf*^{V600E}/*Pten*$^{-/-}$ *M*ice

Veronika Morozova[1], Daniele Pellegata[1], Roch-Philippe Charles[1], Jürg Gertsch[1]

[1]Institute of Biochemistry and Molecular Medicine, University of Bern, Switzerland

* Correspondence: juerg.gertsch@unibe.ch

LC-ESI-MS/MS conditions for amino acids and neurotransmitters

The internal standards used for LC-ESI-MS/MS analysis: Ethanolamine_d4, Dopamine_d4, Methionine_d3, Serine_d3, Lysine_d4 Glycine_d5 were purchased from Eurisotop France, Acetylcholine_d4 was purchased from Sigma Aldrich. The list of the internal standards and their corresponding analytes are listed in Supplementary Table S1. The gradient elution profile used is detailed in Supplementary Table S2. The flow rate was 1.00 ml/min. The Multiple Reaction Monitoring (MRM) transitions monitored for quantification of the analytes are listed in Supplementary Table S3.

LC-ESI-MS/MS conditions for endocannabinoids and bioactive lipids

The internal standards used for LC-ESI-MS/MS analysis: 2-AG-d5, LEA-d4, OEA-d4, PEA-d5, Corticosterone-d4, ARA-d8, PGE2-d4 were purchased from Eurisotop France. The list of the internal standards and their corresponding analytes are listed in Supplementary Table S1. The gradient elution profiles used for positive and negative modes are detailed in Supplementary Tables S4 and S5. The flow rate was 350 µl/min for positive mode and 300 µl/min for negative mode. The Multiple Reaction Monitoring (MRM) transitions monitored for quantification of the analytes are listed in Supplementary Tables S6 for positive mode, S7 for negative mode and S8 for internal standards.

Supplementary Table S1: LC-ESI-MS/MS internal standards and covered analytes.

Internal Standard	Analytes
Ethanolamine-d4	ETA, GABA, GSH, Ado
Methionine-d3	Met, Trp, Tyr, Phe, Leu, Val, Ala, Tau
Serine-d3	Ser, Glu, Gln, Asp, Asn
Lysine-d4	Lys, Arg, His
Glycine-d5	Gly, Pro, Cys
Acetylcholine-d4	Ach, Ch
Dopamine-d4	5-HT, DA, L-DOPA, NE
2-AG-d5	2-AG, 2-OG, SAG, C18-20:4 PC, C18-20:4 PE, 20:4 PE
AEA-d4	AEA
LEA-d5	LEA
Corticosterone-d4	Corticosterone
PEA-d4	PEA, SEA
OEA-d4	OEA
ARA-d8	ARA
PGE2-d4	PGE2, PGD2

Supplementary Table S2: LC-ESI-MS/MS gradient elution profile for quantification of the amino acid and neurotransmitter analytes.

Time (min)	% Aqueous Phase A
0.01	8
3.00	12
6.40	30
6.50	100
10.00	100
10.10	8
12.90	8

Supplementary Table S3: Multiple Reaction Monitoring (MRM) transitions monitored for quantification of the amino acid and neurotransmitter analytes LC-ESI-MS/MS.

Analyte	Precursor Ion (m/z)	Product Ion (m/z)	Polarity
Ach	146.10	87.10	Positive
Leu	132.10	86.10	Positive
Lys	147.10	84.10	Positive
Phe	166.10	120.30	Positive
GSH	308.20	179.10	Positive
ETA	62.00	44.00	Positive
Arg	175.10	69.90	Positive
Cys	122.10	76.10	Positive
Val	118.00	72.10	Positive
Ala	90.00	44.00	Positive
Tyr	182.10	136.20	Positive
Ado	268.20	136.20	Positive
Gly	76.00	30.10	Positive
His	156.10	110.10	Positive
Met	150.10	104.20	Positive
Tau	126.00	108.00	Positive
GABA	104.00	87.00	Positive
Pro	116.00	70.00	Positive
Ser	106.00	60.00	Positive
Trp	205.10	188.10	Positive
Ch	104.10	60.10	Positive
Asn	133.00	73.90	Positive
Asp	134.00	74.00	Positive
Glu	148.10	84.00	Positive
Gln	147.10	130.10	Positive

Supplementary Table S4: Positive mode LC-ESI-MS/MS gradient elution profile for endocannabinoids and bioactive lipids.

Time (min)	% Aqueous Phase
0.50	85
3.50	30
8.00	1
12.00	1
13.00	85
15.50	85

Supplementary Table S5: Negative mode LC-ESI-MS/MS gradient elution profile for endocannabinoids and bioactive lipids.

Time (min)	% Aqueous Phase
3.00	60
9.00	35
10.00	5
14.00	5
15.00	95
17.00	95

Supplementary Table S6: Multiple Reaction Monitoring (MRM) transitions monitored for quantification of endocannabinoids and bioactive lipids during LC-ESI-MS/MS in positive mode.

Analyte	Precursor Ion (m/z)	Product Ion (m/z)		Polarity
2-AG	379.00	203.00	287.00	Positive
AEA	348.00	62.00	133.00	Positive
OEA	326.00	62.00	309.00	Positive
LEA	324.00	62.00	109.00	Positive
PEA	300.00	62.00	283.00	Positive
SEA	328.00	62.00	311.00	Positive
2-OG	357.30	265.30	247.40	Positive
NE	365.00	133.00	121.00	Positive
Corticosterone	347.00	121.00	97.00	Positive
Cortisol	363.00	97.00	121.00	Positive

SAG	645.4	341.10	327.10	Positive
C18(Plasm)-20:4 PC	794.60	184.00	86.10	Positive
C18(Plasm)-20:4 PE	752.60	361.40	392.40	Positive
20:4 PE	788.50	44.10	647.60	Positive

Supplementary Table S7: Multiple Reaction Monitoring (MRM) transitions monitored for quantification of endocannabinoids and bioactive lipids during LC-ESI-MS/MS in negative mode.

Analyte	Precursor Ion (m/z)	Product Ion (m/z)		Polarity
ARA	303.00	59.00	259.00	Negative
PGE2	351.00	271.00	315.20	Negative
PGD2	351.00	189.10	319.00	Negative

Supplementary Table S8: Multiple Reaction Monitoring (MRM) transitions monitored for quantification of the endocannabinoid and bioactive lipid internal standards during LC-ESI-MS/MS.

Internal Standard	Precursor Ion (m/z)	Product Ion (m/z)	Polarity
2-AG-d5	384.00	287.00	Positive
AEA-d4	352.00	66.00	Positive
LEA-d5	328.00	66.00	Positive
Corticosterone-d4	367.00	121.00	Positive
PEA-d4	305.00	62.00	Positive
OEA-d4	330.00	66.00	Positive
ARA-d8	311.00	59.00	Negative
PGE2-d4	355.00	319.00	Negative

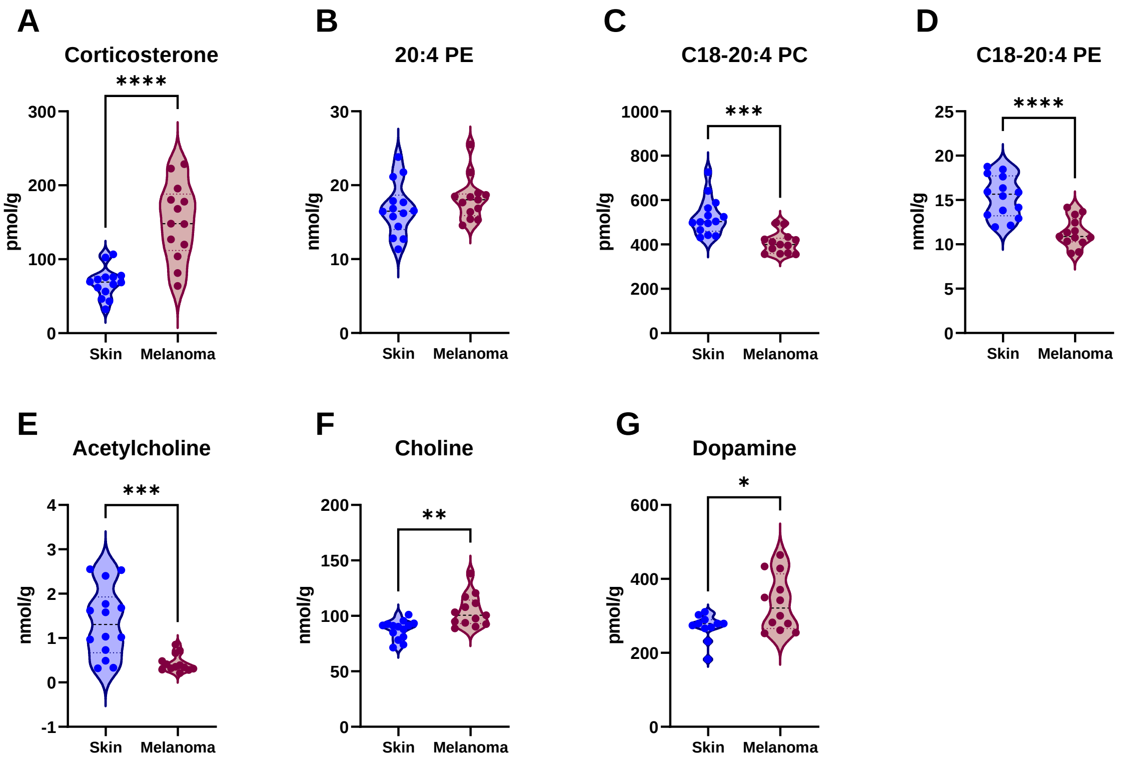

Supplementary Figure S1. Endogenous bioactive lipid and neurotransmitter levels in mouse melanoma and skin. (A-G) Data are shown as violin plots (N = 14 for each group) and analysed by two-tailed paired t-test; *P < 0.05; **P < 0.01; ***P < 0.001; ****P < 0.0001. Abbreviations: 20:4 PE , 1,2-diarachidonoyl-sn-glycero-3-phosphoethanolamine; C18-20:4 PC, 1-(1Z-octadecenyl)-2-arachidonoyl-sn-glycero-3-phosphocholine; C18-20:4 PE, 1-(1Z-octadecenyl)-2-arachidonoyl-sn-glycero-3-phosphoethanolamine.

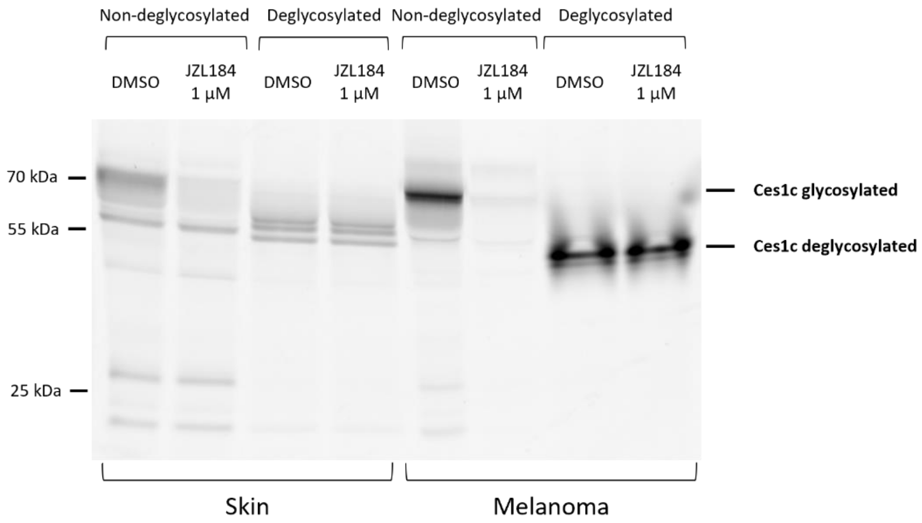

Supplementary Figure S2. Representative gel-based ABPP of deglycosylated mouse skin samples. After the deglycosylation process, samples were incubated with the activity probe TAMRA-FP (125 nM) for 30 min at RT.

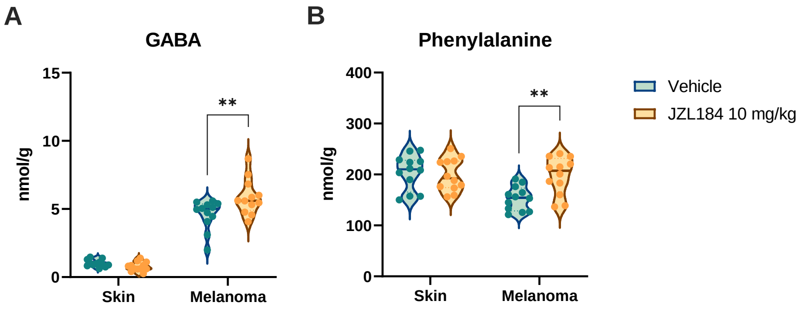

Supplementary Figure S3. Effect of JZL184 *in vivo*. (A,B) Representative biomolecules influenced by the JZL184 treatment *in vivo*. Data are shown as violin plots (N = 12 for each group) and analysed by two-way ANOVA with multiple comparisons and Tukey's post hoc test; **P <0.01. Abbreviations: GABA, gamma-aminobutyric acid.

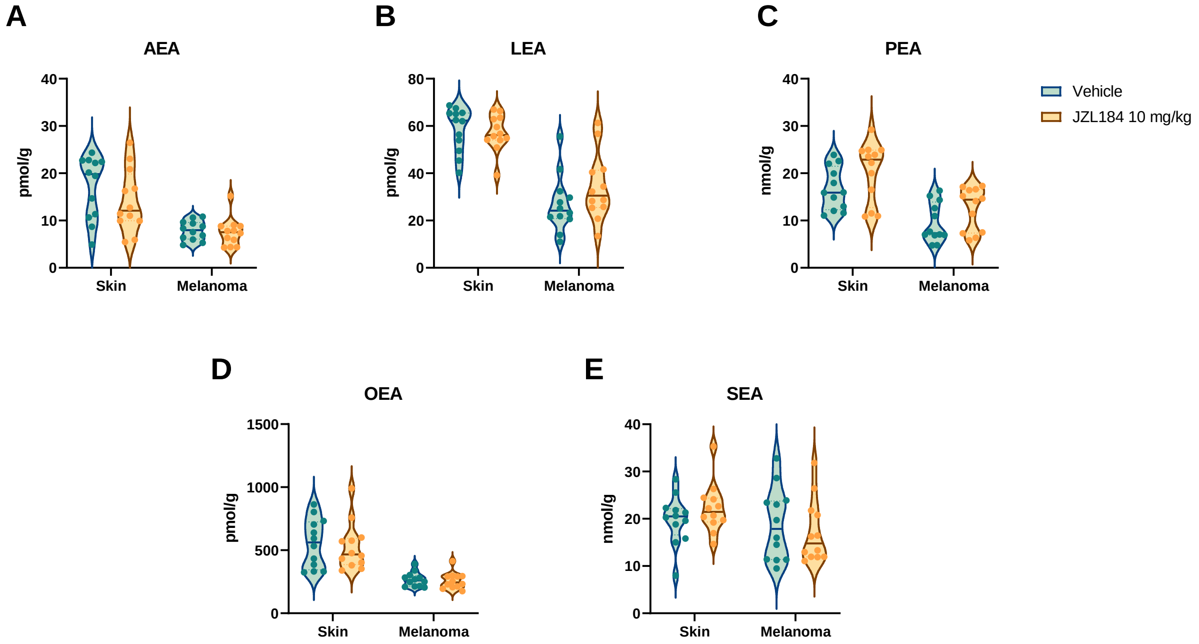

Supplementary Figure S4. Effect of JZL184 *in vivo* on N-Acylethanolamines (NAEs). (A-D) Representative biomolecules influenced by the JZL184 treatment *in vivo*. Data are shown as violin plots (N = 12 for each group) and analysed by two-way ANOVA with multiple comparisons and Tukey's post hoc test. Abbreviations: AEA, N-Arachidonoyl-ethanolamine, Anandamide; LEA, N-Linoleoyl-ethanolamine; OEA, N-oleoyl-ethanolamine; PEA, N-palmitoyl-ethanolamine; SEA, N-stearoyl-ethanolamine.

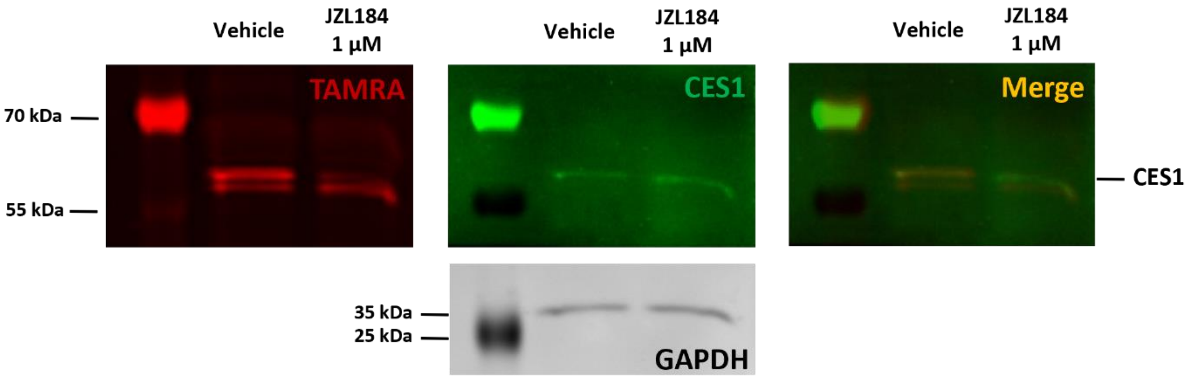

Supplementary Figure S5. Representative gel-based ABPP and immunoblot overlay of human skin. Samples were first incubated with the activity probe TAMRA-FP (125 nM) (red) and then blotted with anti-CES1 antibody (green). GAPDH was used as loading control.

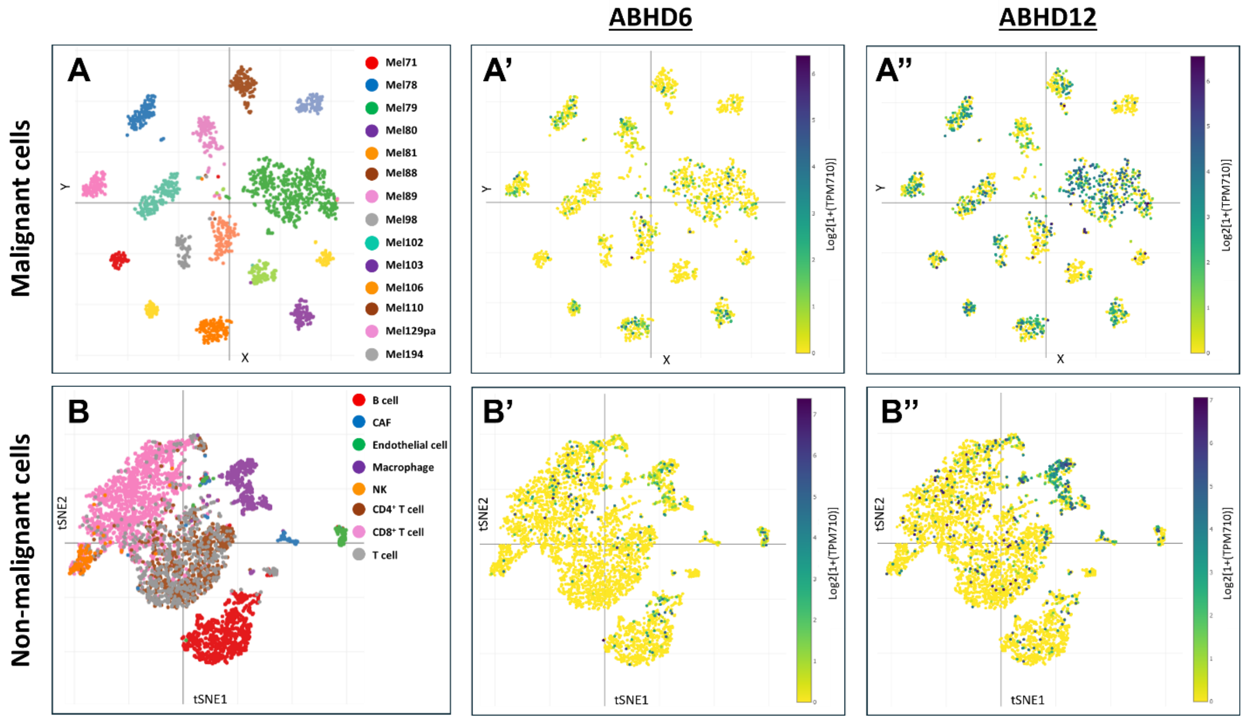

C

	CES1	MAGL	ABHD6	ABHD12
Mel71	0	0	0.05	0.78
Mel78	0.55	0.02	0.51	1.33
Mel79	0	0.22	0.29	1.85
Mel80	0.09	0.54	0.57	1.00
Mel81	0.01	1.02	0.53	1.21
Mel88	0.16	0.38	0.12	0.81
Mel89	0.02	0.11	0.40	1.01
Mel98	0	0.40	0.10	1.18
Mel102	0.02	0.01	0.09	0.83
Mel103	0	0.10	0.29	0.69
Mel106	0.01	0.14	0.02	0.14
Mel110	0.01	0.17	0.35	0.53
Mel129pa	0	0.13	0.01	0.28
Mel194	0	0.23	0.28	1.05

D

	CES1	MAGL	ABHD6	ABHD12
Macrophage	0.11	1.39	0.32	1.33
Endothelial cell	0.04	1.90	0.42	0.60
CAF	0.21	1.17	0.14	0.91
CD4+ T cell	0.01	0.02	0.08	0.30
CD8+ T cell	0.01	0.05	0.16	0.27
T cell	0.02	0.10	0.07	0.20
NK	0.05	0.09	0.05	0.15
B cell	0	0.11	0.17	0.17

(Colour scale: 0–2)

Supplementary Figure S6. ABHD6, ABHD12, and FAAH expression in human melanoma. Scatter plots of the integrative analysis of single-cell RNA sequencing (scRNA-seq) data of human melanoma samples with a t-stochastic neighbour embedding (t-SNE) of single-cell profiles (dots) of (A) malignant and (B) non-malignant cells, coloured by (A) tumour or by (B) post hoc annotation. (A) Only tumours with at least 50 malignant cells are shown. Expression of ABHD6 in (A') malignant and (B') non-malignant cells; and expression of ABHD12 in (A'') malignant and (B'') non-malignant cells. Heatmaps depicting CES1, MAGL, ABHD6 and ABHD12 expression in (C) malignant and (D) non-malignant cells. Red colour indicates positive expression (> 0), white colour indicates zero (0), and the colour intensity represents variation in the levels across the colour scale. Abbreviations: CAF, cancer-associated fibroblast NK, natural killer cell (1,2).

References

1. Tirosh I, Izar B, Prakadan SM, Wadsworth MH, Treacy D, Trombetta JJ, et al. Dissecting the multicellular ecosystem of metastatic melanoma by single-cell RNA-seq. Science. 2016;352(6282):189–96.
2. Jerby-Arnon L, Shah P, Cuoco MS, Rodman C, Su MJ, Melms JC, et al. A Cancer Cell Program Promotes T Cell Exclusion and Resistance to Checkpoint Blockade. Cell. 2018;175(4):984-997.e24.
